# Ketogenic diet combined with probiotics for polyendocrine metabolic ovarian syndrome: research progress from the perspective of the gut microbiota–host metabolic axis

**DOI:** 10.3389/fnut.2026.1945464

**Published:** 2026-09-02

**Authors:** Yi Yang, Furen Dai, Xiaoming He, Pingping Zhao, Qiuxia Chen

**Affiliations:** 1 The Second Affiliated Hospital of Zhejiang Chinese Medical University, Hangzhou, China; 2 The Third Affiliated Hospital of Wenzhou Medical University, Wenzhou, China

**Keywords:** gut microbiota, insulin resistance, ketogenic diet, polyendocrine metabolic ovarian syndrome, probiotics

## Abstract

Polyendocrine metabolic ovarian syndrome (PMOS), formerly known as polycystic ovary syndrome (PCOS), is a common endocrine-metabolic disorder characterized by insulin resistance, androgen excess, chronic inflammation, and gut microbial dysbiosis. Increasing evidence suggests that the gut microbiota–metabolite–host axis contributes to the interaction between metabolic dysfunction, immune imbalance, and reproductive endocrine abnormalities. The ketogenic diet (KD) may improve PMOS by restricting carbohydrate intake, promoting ketone production, reducing body weight, enhancing insulin sensitivity, and alleviating inflammation. However, KD may also decrease beneficial bacteria, including *Bifidobacterium*, potentially disrupting intestinal microbial homeostasis. Probiotics may counteract these effects by restoring beneficial microbial populations, increasing short-chain fatty acid production, strengthening the intestinal barrier, regulating bile acid metabolism, and reducing systemic inflammation. These changes may further improve glucose and lipid metabolism and reproductive endocrine function. This review summarizes the potential mechanisms and current evidence regarding the combined use of KD and probiotics in PMOS, with particular emphasis on the gut microbiota–host metabolic axis. However, no randomized controlled trial has directly evaluated this combined approach in women with PCOS. Therefore, its potential complementary effects remain hypothetical and require clinical validation.

## 1 Introduction

### 1.1 Epidemiological burden and clinical challenges of PMOS

#### 1.1.1 Global and Chinese disease burden of PMOS

Polyendocrine metabolic ovarian syndrome (PMOS)—formerly known as polycystic ovary syndrome (PCOS)—is the most common endocrine-metabolic disorder among women of reproductive age (1). Although polycystic ovary syndrome (PCOS) remains the internationally accepted term, the term polyendocrine metabolic ovarian syndrome (PMOS) is used in this review to emphasize that the condition involves interacting reproductive, endocrine, and metabolic abnormalities and is not limited to ovarian morphology. To avoid terminological ambiguity, PCOS is retained when referring to established diagnostic criteria, clinical guidelines, and the terminology used in the original studies. Its clinical manifestations include elevated androgen levels, menstrual irregularities, and polycystic ovarian morphology, often accompanied by systemic metabolic alterations such as insulin resistance, obesity, dyslipidemia, and chronic low-grade inflammation (2). The development of PCOS is associated with disturbances in the hypothalamic–pituitary–ovarian axis, insulin resistance, and compensatory hyperinsulinemia. Elevated insulin levels can stimulate ovarian androgen production, further impair ovulation and worsen metabolic dysfunction. These changes interact with one another, forming a vicious cycle between reproductive endocrine abnormalities and metabolic imbalance.

Epidemiological studies indicate that the global prevalence of PCOS among women of reproductive age ranges from approximately 5 to 20% (roughly 15–20% by Rotterdam criteria and 6–10% by National Institutes of Health criteria) (3). With the rising prevalence of obesity, insulin resistance, and lifestyle-related metabolic abnormalities, the disease burden associated with PCOS is increasing. Studies in Chinese community populations show a prevalence of approximately 5.6–7.8%, with obese PCOS accounting for over 50% of cases and the total patient population exceeding 30 million (4). Furthermore, PCOS remains underdiagnosed and undertreated; although most patients initially seek care at gynecology clinics due to menstrual irregularities, clinical recognition rates remain notably low, with only about 30% receiving a definitive diagnosis at an early stage (2).

Regarding long-term health risks, PCOS has evolved beyond a traditional reproductive-endocrine disorder, presenting a complex burden that spans metabolic, reproductive, and oncological domains. Research indicates that women with PCOS face a risk of type 2 diabetes that is 3–5 times higher than that of the general population, along with a 40–50% increased risk of gestational diabetes (5). Additionally, the risk of cardiovascular disease is 1.5–2 times higher, and the risk of endometrial cancer is 2–6 times higher. Moreover, as a major cause of infertility due to ovulatory dysfunction, PCOS significantly impacts female fertility. Collectively, the aforementioned evidence indicates that the health impact of PCOS extends far beyond menstrual irregularities or infertility; rather, it is a systemic public health issue that urgently requires a comprehensive management model based on multidisciplinary collaboration.

#### 1.1.2 Limitations of existing therapeutic strategies

Despite the significant disease burden associated with PCOS, current treatments primarily focus on symptom control and struggle to simultaneously address the multiple pathological components—such as reproductive, metabolic, and inflammatory processes. While combined oral contraceptives can regulate the menstrual cycle and lower androgen levels, they offer limited improvement for insulin resistance and metabolic abnormalities, and long-term use may increase the risk of thrombosis (6). Metformin, an insulin-sensitizing agent, improves insulin sensitivity and glucose tolerance; however, gastrointestinal side effects lead to poor tolerability and reduced adherence in some patients, and its efficacy in improving reproductive function remains inconsistent (7). Anti-androgen agents, such as spironolactone, are commonly used to relieve symptoms associated with androgen excess. However, because of their potential teratogenic effects, reliable contraception is required during treatment (2). Ovulation induction with clomiphene may improve fertility in some patients, although it is also associated with an increased risk of ovarian hyperstimulation and multiple pregnancy.

Lifestyle modification, including dietary changes, regular physical activity, and behavioral support, is generally recommended as the first-line approach to PCOS management. In clinical practice, however, long-term adherence remains a major challenge (8). Fewer than 20% of patients have been reported to maintain a structured lifestyle program for longer than 6 months (9). In addition, dietary advice that focuses mainly on calorie restriction may not fully account for the metabolic differences among patients with PCOS, such as varying degrees of insulin resistance, hyperandrogenism, and inflammation. This may partly explain the inconsistent responses observed across different individuals.

Although current pharmacological and lifestyle-based treatments can improve specific symptoms, their effects are often limited to particular aspects of the disorder. As a result, they may not adequately address the combined reproductive, metabolic, and inflammatory abnormalities involved in PCOS.

#### 1.1.3 Gut microbiota: from associated factor to intervention target

To overcome the limitations of current PCOS interventions, there is an urgent need for innovative strategies that are cost-effective, operate via clear mechanisms, and are suitable for large-scale application. The human gut harbors approximately 3.8 × 10^13^ microorganisms (10), constituting the host’s largest symbiotic ecosystem. Gut microbiota play a pivotal role in regulating host energy metabolism, immune homeostasis, and endocrine function (11). Existing research consistently demonstrates that gut microbiota dysbiosis contributes to disease progression by compromising intestinal barrier integrity, inducing chronic low-grade inflammation, and disrupting metabolic homeostasis. Mechanistically, altered microbial composition promotes the development of PCOS phenotypes through multiple pathways: first, a reduction in SCFA-producing commensal bacteria (such as *Faecalibacterium* and *Roseburia*) impairs intestinal barrier repair and diminishes systemic anti-inflammatory signaling; second, the overgrowth of Gram-negative bacteria (such as *Bacteroides* and *Prevotella*) increases lipopolysaccharide (LPS) production—LPS translocates across the compromised intestinal barrier into the bloodstream (12), activating the toll-like receptor 4 (TLR4)/nuclear factor kappa B (NF-κB) inflammatory pathway and further exacerbating systemic inflammation and insulin resistance; finally, dysregulated microbial control over BA metabolism, the estrogen enterohepatic circulation (estrobolome) (13), and branched-chain amino acid (BCAA) metabolism further aggravates insulin resistance and androgen excess. Together, these mechanisms suggest that interactions among the gut microbiota, host metabolism, and immune responses may contribute to the development of PCOS. Evidence from animal studies further supports this association. Fecal microbiota transplantation from women with PCOS to germ-free mice has been shown to induce metabolic and reproductive abnormalities resembling those observed in PCOS (14). In contrast, transplantation of microbiota from healthy donors into PCOS model mice can improve insulin sensitivity, ovulatory function, and hormonal balance. These findings indicate that restoring the composition and function of the gut microbiota may offer a potential approach to improving the metabolic and reproductive disturbances associated with PCOS.

Several gut-related mechanisms implicated in PCOS, including impaired intestinal barrier function, increased LPS translocation, and activation of the TLR4/NF-κB signaling pathway, are also involved in immune-mediated gastrointestinal disorders such as inflammatory bowel disease and celiac disease. This overlap suggests that intestinal immune dysfunction may contribute not only to local gastrointestinal inflammation but also to systemic metabolic and reproductive disturbances. Accordingly, nutritional strategies that regulate intestinal inflammation and immune activity may also have potential value in the management of PCOS.

### 1.2 KD: a systematic intervention strategy

#### 1.2.1 The KD and its metabolic basis

The KD is a dietary pattern characterized by high fat, moderate protein and very low carbohydrate intake. It was first proposed by American doctor Wilder in 1921 and was originally used to simulate a starvation state to treat refractory epilepsy (15). When carbohydrate intake is significantly reduced, liver glycogen reserves are usually gradually depleted within 24 to 48 h. In order to maintain energy supply, the body begins to mobilize triglycerides in adipose tissue and release large amounts of free fatty acids through lipolysis (16). After these fatty acids enter the liver, they are β-oxidized to produce acetyl-CoA, which is further synthesized into ketone bodies such as β-hydroxybutyrate (β-HB), acetoacetate (AcAc), and acetone.

In the past decade, evidence of the application of KD in obesity, type 2 diabetes, and metabolic syndrome has been accumulating. Its metabolic benefits are not from a single source (17), but from synergistic effects at multiple levels: low carbohydrate intake directly weakens postprandial blood glucose and insulin peaks, improving insulin sensitivity; high-fat diet stimulates the secretion of satiety hormones such as glucagon-like peptide-1 (GLP-1) and peptide YY (PYY), thereby reducing total energy intake; in addition, the ketone body β-HB itself can inhibit the NOD-like receptor family pyrin domain-containing 3 (NLRP3) inflammasome, regulate gene expression, and participate in the regulation of energy metabolism as a ligand of free fatty acid receptor 3 (FFAR3)/G protein-coupled receptor 41 (GPR41) (18). These multi-target effects make KD go beyond the traditional weight loss diet category and demonstrate the potential of systemic metabolic intervention.

Further molecular mechanism research revealed the key to KD weight loss. Lu et al. found that KD can promote the transcription and secretion of growth differentiation factor 15 (GDF15) by activating hepatic peroxisome proliferator-activated receptor gamma (PPARγ) (19). The circulating GDF15 acts on the glial cell line-derived neurotrophic factor family receptor alpha-like (GFRAL) receptor in the brainstem, suppressing appetite and increasing energy consumption. In GDF15 and its receptor GFRAL gene knockout mice, the weight loss effect of KD completely disappeared, clarifying the key role of the GDF15-GFRAL signaling pathway in KD-mediated weight regulation. This discovery not only improves the metabolic regulatory network of KD, but also provides new molecular targets for its application in metabolic diseases.

#### 1.2.2 Preliminary evidence for the KD in PCOS

The theoretical basis for incorporating the KD into PCOS management lies in the high degree of alignment between its intervention targets—insulin resistance, obesity, and chronic low-grade inflammation—and the core pathophysiology of PCOS. A prospective study by Paoli et al. demonstrated that a 12-week low-calorie ketogenic diet (LCKD) intervention in 14 overweight women with PCOS resulted in an average weight loss of 9.5 kg, a reduction of approximately 50% in the homeostatic model assessment of insulin resistance (HOMA-IR) (20), and a significant decrease in total testosterone levels; notably, 50% of the patients regained regular menstrual cycles. Subsequent randomized controlled trials and prospective studies have also reported beneficial effects of the ketogenic diet on body weight, glucose metabolism, and hormonal profiles in women with PCOS (21). However, most clinical studies have mainly examined the relationship between dietary intervention and changes in clinical outcomes. The mechanisms linking diet, gut microbiota, microbial metabolites, and host responses have received comparatively less attention. It is also worth noting that β-hydroxybutyrate (β-HB), a major ketone body produced during a ketogenic diet, may suppress the growth of *Bifidobacterium* and reduce its relative abundance in the gut. This finding raises the possibility that a ketogenic diet, despite its metabolic benefits, may also have unfavorable effects on certain beneficial gut bacteria.

### 1.3 Probiotic intervention targeting the gut microbiota

Probiotics are defined as live microorganisms that confer health benefits to the host when administered in adequate amounts (22). They can inhibit the growth of pathogenic bacteria, produce bioactive metabolites, modulate the gut environment, and enhance host immune function. The potential value of probiotics in managing PCOS stems primarily from their regulation of the gut microbiota–host axis; specific probiotic strains may restore gut microbial balance and support SCFA-producing bacterial communities (23). Furthermore, probiotics inhibit the growth of pro-inflammatory Gram-negative bacteria through competitive exclusion, thereby reducing LPS levels. Additionally, probiotic metabolites (such as SCFAs and bacteriocins) act as signaling molecules that remotely regulate metabolic functions in the liver, adipose tissue, and ovaries (24). Although studies have explored the potential impact of probiotic interventions on the metabolic phenotype of PCOS, significant variations across trials regarding strain composition, intervention duration, and baseline participant characteristics have resulted in heterogeneous findings.

Existing research has provided preliminary evidence supporting probiotic interventions for PCOS. A meta-analysis by Li et al., encompassing 17 RCTs and 1,049 participants, demonstrated that probiotics, prebiotics, and synbiotics could significantly reduce fasting blood glucose, HOMA-IR, triglyceride, and total cholesterol levels in patients with PCOS (25). In animal studies, He et al. showed that *Lactiplantibacillus plantarum* CCFM1019 alleviated symptoms in letrozole-induced PCOS rats via a butyrate-dependent gut–brain axis mechanism; the treatment restored testosterone and luteinizing hormone (LH) levels and increased the abundance of SCFA-producing bacteria such as *Lachnospira* and *Ruminococcus* (26). While animal studies have provided mechanistic insights, the comparability and reproducibility of results in clinical studies remain limited due to variations in strains, dosages, treatment durations, and assessment metrics. More importantly, there is currently no research systematically evaluating the synergistic effects of a “KD combined with probiotics” in PCOS. This fragmented landscape—where both dietary and microbiota-based interventions are known to be effective, yet the combined strategy remains unexplored—highlights a research gap regarding the mechanisms and optimization of such combined interventions. Therefore, systematically elucidating the mechanisms underlying this combined intervention will help provide new approaches for the health management of PCOS.

### 1.4 Objectives of this review

The effects of KD and probiotic interventions on PCOS involve the interplay among metabolism, gut microbiota, and inflammation. Both exert systemic regulatory effects via the “gut microbiota–metabolite–host” axis, yet they operate through distinct mechanisms: KD primarily remodels the substrate environment for microbiota at the metabolic level, whereas probiotics directly supplement functional microbial populations at the microecological level. This review aims to systematically integrate evidence from animal experiments and clinical studies to elucidate the characteristics and mechanisms of gut microbiota dysbiosis in PCOS. It further analyzes the multi-level pathways through which KD ameliorates PCOS by modulating the microbiota, thereby providing a basis for decision-making regarding precision interventions that combine KD and probiotics—encompassing dietary regimen design, strain selection, and synergistic strategies with conventional therapies.

### 1.5 Literature search and study selection

This study was conducted as a semi-systematic narrative review to synthesize clinical, animal, and mechanistic evidence concerning ketogenic diets, probiotics, and the gut microbiota in PMOS/PCOS.

#### 1.5.1 Search strategy

A structured literature search was conducted in PubMed, Web of Science, Cochrane Library, ScienceDirect, and CNKI from database inception to 1 May 2026. The search terms included (“polyendocrine metabolic ovarian syndrome” OR “polycystic ovary syndrome” OR “PMOS” OR “PCOS”) AND (“ketogenic diet” OR “low-carbohydrate diet” OR “nutritional ketosis”) AND/OR (“gut microbiota” OR “gut microbiome” OR “probiotic” OR “probiotics” OR “prebiotic” OR “synbiotic”). Reference lists of relevant reviews and original studies were also manually screened.

#### 1.5.2 Eligibility criteria

Eligible studies included clinical studies involving women with PMOS/PCOS and animal studies using established PCOS models. Studies were required to evaluate ketogenic diets, probiotics, or microbiota-related interventions and report metabolic, endocrine, reproductive, inflammatory, intestinal barrier, or gut microbiota outcomes.

Reviews, meta-analyses, clinical trials, observational studies, and controlled animal experiments were considered. Conference abstracts, editorials, duplicate publications, studies unrelated to PMOS/PCOS, and articles without accessible full text or sufficient outcome data were excluded.

#### 1.5.3 Study selection and data extraction

Two authors independently screened titles, abstracts, and full texts. Extracted information included study design, sample characteristics, intervention type, probiotic strains and dosage, dietary regimen, intervention duration, and major metabolic, hormonal, reproductive, inflammatory, and gut microbiota outcomes. Disagreements were resolved through discussion.

## 2 PCOS and gut microbiota dysbiosis: from association to causality

### 2.1 Characteristic alterations of gut microbiota in patients with PCOS

Dysbiosis of the gut microbiota has become a recognized characteristic in patients with PCOS. Li et al. quantified this feature at the population level through a large-scale systematic review and meta-analysis involving 28 case–control studies and 1,950 subjects (27); the results revealed a simultaneous decline in gut microbiota evenness and phylogenetic diversity, indicating structural degradation of the gut ecosystem at the evolutionary lineage level. While metrics reflecting species richness—such as “Observed species” and Chao1—showed a downward trend in most studies, some heterogeneity remained across different studies. Another age-matched case–control study similarly confirmed a significant reduction in gut microbiota richness and alpha diversity in PCOS patients; beta-diversity analysis also demonstrated distinct separation of community structures between groups, indicating altered community composition patterns in PCOS (28). The reduced microbial diversity observed in PCOS may indicate impaired stability of the gut ecosystem. This disturbance could make the microbiota less resistant to external changes and may also influence intestinal metabolism, inflammatory activity, and host immune function.

Against the backdrop of overall reduced diversity, the abundance of gut microbiota at the phylum, family, and genus levels undergoes systematic remodeling in PCOS patients; specifically, the abundance of the two major phyla—Firmicutes and Bacteroidota—is significantly reduced, whereas that of Actinobacteriota and Proteobacteria is significantly increased (28). Consistent alterations in specific genera further delineate the microbial characteristics of PCOS; multiple studies indicate a reduction in SCFA-producing bacteria and BA-metabolizing bacteria, alongside an increase in certain genera associated with inflammatory responses or metabolic disorders. Clinical evidence shows significantly elevated levels of *Parabacteroides merdae*, *Bacteroides fragilis*, and *Escherichia/Shigella* in the gut of PCOS patients, whereas *Faecalibacterium prausnitzii* is relatively more abundant in healthy controls (29). Further analysis reveals that these microbial changes correlate positively with body mass index (BMI), serum testosterone, LH, and anti-Müllerian hormone (AMH) levels, suggesting that gut microbiota dysbiosis may be closely linked to the metabolic and reproductive phenotypes of PCOS. Through an integrated analysis of 16S ribosomal RNA (16S rRNA) gene sequencing and serum metabolomics, Yu et al. further confirmed that gut microbiota alpha-diversity was significantly lower in the PCOS group compared to healthy controls (30); genera such as *Escherichia/Shigella* and *Alistipes* were significantly more abundant in the PCOS group, while several beneficial bacterial groups, such as *Roseburia*, were relatively reduced. Furthermore, studies on normal-weight PCOS patients have shown that the subgroup with insulin resistance exhibits a significant increase in *Rothia*, *Ruminococcus*, and *Enterococcus*, alongside a relative decrease in *Prevotella*; these alterations correlate with markers of insulin resistance, suggesting that changes in the abundance of specific bacterial taxa may be directly linked to metabolic phenotypes (31). In animal models, Qi et al. identified a gut microbiota–BA–IL-22 regulatory axis; they observed significant enrichment of *Bacteroides vulgatus* in PCOS patients (32), accompanied by altered gut bile acid profiles and reduced IL-22 levels. Subsequent fecal microbiota transplantation (FMT) from PCOS patients—or from recipient mice colonized with *B. vulgatus*—into recipient mice induced insulin resistance, ovarian dysfunction, and chronic inflammation, whereas supplementation with specific BAs or IL-22 partially ameliorated these pathological phenotypes, indicating a direct link between the microbiota and metabolic and immune signaling pathways.

Gut microbiota dysbiosis in PCOS is characterized not only by reduced alpha diversity and altered community structure but also by distinct patterns corresponding to clinical phenotypes such as hyperandrogenism, insulin resistance, and obesity. Miao et al. found that gut microbiota diversity was significantly lower in PCOS patients with hyperandrogenism and that the abundance of specific bacteria correlated with serum steroid levels (33). Compared to patients with low androgen levels, those with hyperandrogenism exhibited different structural changes in the microbiota (e.g., increased *Prevotella* and decreased *Faecalibacterium*). Collectively, this evidence suggests a close and complex interplay between gut microbiota composition and host metabolism, inflammation, and hormonal homeostasis in PCOS. It should be noted, however, that the direction of abundance changes for certain bacteria varies across studies; this inconsistency may stem from factors such as the subjects’ BMI, dietary patterns, geographic differences, and statistical methodologies. Therefore, when characterizing the gut microbiota in PCOS, it is more appropriate to emphasize the trend of “overall microbial dysbiosis” rather than absolute changes in specific bacterial genera. This pattern of dysbiosis is generally characterized by reduced diversity and altered SCFA production. There is a decrease in bacterial abundance alongside a remodeling of bile acid metabolism and a trend toward the enrichment of pro-inflammatory and Gram-negative bacteria. This structural reorganization at the phylum and genus levels indicates that the gut microbiota of PCOS patients undergoes not only a reduction in species diversity but also a shift in functional bacterial communities; such shifts may influence host energy metabolism, immune status, and endocrine regulation through their metabolites (e.g., SCFAs, LPS).

### 2.2 Causal evidence linking gut microbiota to PCOS

Although numerous population-based studies have demonstrated a significant association between gut microbiota dysbiosis and clinical phenotypes in patients with PCOS, a central question in the field remains whether these microbial alterations are merely concomitant phenomena or play a causative role in the disease. In recent years, researchers have employed Mendelian randomization (MR) analysis and FMT experiments—spanning both human genetics and animal models—to evaluate the involvement of gut microbiota in the pathogenesis and progression of PCOS.

The MR approach treats gut microbiota as the exposure variable and PCOS as the outcome variable, using genetic instrumental variables to reduce, rather than completely eliminate, the influence of confounding and reverse causation. Sun et al., leveraging large-scale genome-wide association study (GWAS) data comprising tens of thousands of cases and controls, identified eight gut microbiota taxa showing statistically significant genetically predicted associations with PCOS risk (34). Among these, genera such as *Streptococcus*, *Ruminococcaceae* UCG005, and *Actinomyces* emerged as risk factors for PCOS in the MR analysis, whereas others, such as *Sellimonas*, appeared to exert a protective effect. Conversely, reverse MR analysis did not identify statistically significant evidence that genetically predicted PCOS liability affects the gut microbiota. However, this null finding should not be interpreted as proof that PCOS has no effect on microbial composition. Additionally, a separate bidirectional MR study provided complementary evidence (35). Previous research indicated that *Streptococcus* and *Enterorhabdus* might be associated with a reduced risk of PCOS, while taxa such as Tenericutes, *Anaerofilum*, *Coprococcus* 2, *Lachnospiraceae* ND3007, and *Ruminiclostridium* 5 were potentially linked to an increased risk. Mao et al. further identified *Bacilli,* Burkholderiales, and Lachnospiraceae as potential risk-increasing factors, while *Bilophila*, *Blautia*, Cyanobacteria, and *Holdemania* appeared to have protective effects (36). Despite variations in the microbial taxa identified across different MR studies, these findings collectively suggest that certain microbial alterations may be associated with, and potentially contribute to, PCOS susceptibility and progression. However, the specific taxa and directions of effect are not fully consistent across analyses. While MR can infer causality at the population-genetic level, experimentally validating the mechanisms by which gut microbiota contribute to PCOS remains essential. Recent key animal studies utilizing FMT have directly demonstrated that human gut microbiota can transfer PCOS-associated pathological phenotypes. In one such study, fecal samples from PCOS patients were transplanted into germ-free mice (37). Mice receiving the PCOS-derived microbiota exhibited not only a distinct shift in their gut microbial composition compared to the donors but also a suite of PCOS-like pathological features, including weight gain, fat accumulation, significantly exacerbated insulin resistance, elevated serum testosterone, and ovarian ovulatory dysfunction. In contrast, mice receiving fecal microbiota from healthy controls did not display these abnormalities. These results provide experimental evidence that PCOS-associated gut microbiota can contribute to metabolic disturbances and ovarian dysfunction in recipient mice. Further phenotypic analysis revealed that recipient mice developed compromised intestinal barrier integrity, abdominal obesity, and worsened insulin resistance following transplantation. Correlation analyses showed that the abundance of bacterial genera such as *Phocaeicola* and *Mediterraneibacter* was significantly positively correlated with HOMA-IR and blood lipid levels, and statistically associated with endocrine markers, including serum androgens. These findings support a potential contributory role of PCOS-associated gut microbiota in intestinal barrier dysfunction and metabolic and endocrine abnormalities, although the extent to which these effects translate from recipient mice to women with PCOS remains uncertain.

### 2.3 Mechanisms by which gut microbiota influence PCOS

The impact of gut microbiota on PCOS likely results from the combined alteration of microbial metabolic functions, intestinal barrier integrity, and host endocrine regulation. Given that PCOS is a heterogeneous endocrine disorder characterized by significant inter-individual differences in clinical presentation and metabolic abnormalities, gut microbiota dysbiosis exhibits distinct patterns corresponding to specific clinical phenotypes (hyperandrogenism, insulin resistance, and obesity). Microbiota alterations associated with these phenotypes may influence disease progression through distinct mechanisms: the hyperandrogenic phenotype is closely linked to abnormalities in BA metabolism, estrogen enterohepatic circulation, and steroidogenesis regulation; the insulin-resistant phenotype is characterized primarily by disrupted SCFA metabolism and energy dysregulation; and the obese phenotype is frequently associated with intestinal barrier impairment and heightened gut-derived inflammation. Importantly, these relationships are likely bidirectional: microbial alterations may influence host metabolism and endocrine regulation, whereas hyperandrogenism, obesity, and insulin resistance may in turn reshape the gut microbial community. Therefore, the mechanisms discussed below should be interpreted as components of a reciprocal host–microbiota feedback network rather than as strictly unidirectional pathways. Based on these phenotypic differences, gut microbiota likely contribute to the pathogenesis and progression of PCOS primarily through pathways involving SCFA metabolic dysregulation, low-grade inflammation mediated by intestinal barrier damage, and altered sex hormone metabolism.

#### 2.3.1 Hyperandrogenism and microbiota-steroid metabolism

A hyperandrogenic phenotype is one of the most characteristic features of PCOS. Research indicates a link between hyperandrogenism and gut microbiota; the latter can influence the endocrine and metabolic phenotypes of PCOS through mechanisms involving steroid metabolism and SCFA signaling (38). An animal study revealed that mice subjected to prenatal androgenization (PNA) exhibited altered relative abundances of gut bacteria associated with steroid hormone synthesis and SCFA metabolism; specifically, there was an increase in certain steroidogenesis-related bacterial populations, alongside a decrease in genera such as *Akkermansia*, *Bacteroide*s, and *Lactobacillus* (39). Concurrently, PNA mice displayed weight gain, upregulated adipokine expression, and cardiovascular dysfunction. These findings suggest that alterations in gut microbiota structure and metabolic function—particularly involving bacteria capable of steroid transformation, such as *Clostridium scindens*—may contribute to the development of PCOS-related endocrine, metabolic, and cardiovascular abnormalities by modulating host androgen metabolism (40).

#### 2.3.2 SCFA metabolism disorder

Dysregulated SCFA metabolism serves as a crucial link between gut microbiota and the metabolic abnormalities associated with PCOS. SCFAs—primarily acetate, propionate, and butyrate—are produced through the microbial fermentation of indigestible carbohydrates, such as dietary fiber. Beyond serving as an energy source for colonic epithelial cells, they regulate inflammatory responses, insulin sensitivity, and energy metabolism by activating G protein-coupled receptors 41 and 43 (GPR41/GPR43) and inhibiting histone deacetylases (HDACs), thereby indirectly stimulating the secretion of GLP-1 and PYY (41). In patients with PCOS, SCFA-related alterations in the gut microbiota exhibit complex, inconsistent patterns across microbial sources, fecal concentrations, blood levels, and clinical phenotypes. Research by da Silva et al. revealed that women with PCOS display changes in gut microbiota composition, predicted metabolic pathways, and gut-derived metabolites—specifically, reduced levels of indole-3-propionic acid alongside elevated levels of acetate and propionate (42). These alterations correlate with dietary glycemic load and saturated fatty acid intake, suggesting that microbiota-mediated metabolic changes may contribute to the development of PCOS-associated metabolic disorders.

The physiological roles of specific SCFAs differ: butyrate primarily fuels colonic epithelial cells and helps maintain tight junctions and immune homeostasis, generally supporting intestinal barrier integrity and suppressing inflammation; conversely, acetate and propionate are more actively involved in glucose and lipid metabolism as well as enteroendocrine signaling. Their effects are modulated by nutritional status, microbiota composition, and insulin sensitivity; in the context of energy surplus and insulin resistance, altered levels of these SCFAs may reflect aberrant fermentation processes and increased metabolic load.

Furthermore, a reduction in SCFA-producing bacteria or dysregulated SCFA metabolism in PCOS patients compromises the intestinal barrier and the regulation of glucose and lipid metabolism. This leads to insufficient production of GLP-1 and PYY and reduced insulin sensitivity, ultimately progressing to hyperinsulinemia; the hyperinsulinemic state, in turn, stimulates the expression of steroidogenic enzymes—such as cytochrome P450 family 17 subfamily A member 1 (CYP17A1)—in ovarian theca cells, thereby further driving androgen synthesis. Therefore, given the complex bidirectional regulatory relationship between the gut microbiota and host hormone metabolism, targeted regulation of the gut microbiota and its SCFA metabolic pathways holds promise as a potential strategy for ameliorating the hyperandrogenic phenotype and metabolic complications associated with PCOS.

#### 2.3.3 Intestinal barrier damage and low-grade inflammation

Secondly, gut-derived inflammation represents a significant pathway through which the gut microbiota contributes to the pathogenesis and progression of PCOS. Gut microbiota dysbiosis can alter intestinal epithelial tight junctions and barrier integrity, thereby increasing the translocation of microbial components—such as LPS—into the systemic circulation. Once in the circulation, LPS binds to molecules like LPS-binding protein (LBP) and cluster of differentiation 14 (CD14) (43), activating the TLR4/myeloid differentiation primary response 88 (MyD88)/NF-κB signaling pathway and promoting the release of inflammatory cytokines such as tumor necrosis factor-alpha (TNF-α) and interleukin-6 (IL-6); this process establishes persistent, low-grade systemic inflammation. Human studies have provided evidence supporting this mechanism. Zhang et al. found elevated serum zonulin levels in patients with PCOS, correlating with the severity of insulin resistance and ovulatory dysfunction (44); similarly, Zhu et al. observed increased serum LBP levels in PCOS patients, noting a close association with insulin resistance (45). Furthermore, a cohort study of young women with PCOS revealed elevated markers of endotoxemia, suggesting that the entry of gut-derived microbial components into the circulation is not necessarily driven solely by obesity or severe metabolic abnormalities (46). Animal studies have further investigated the link between inflammatory pathways and PCOS phenotypes. In a letrozole-induced PCOS rat model, Wang et al. demonstrated that modulating the gut microbiota suppressed serum LPS levels and ovarian TLR4/NF-κB signaling, reduced inflammatory cytokines (TNF-α, IL-6, and interleukin-8 (IL-8)), and improved body weight, glucose metabolism, androgen levels, and ovarian morphology (47). However, due to heterogeneity across studies regarding population stratification (e.g., BMI matching, age groups) and detection methodologies, it remains impossible to definitively establish a universal, specific “PCOS microbiota signature.” Microbiota dysbiosis and the resulting gut-derived inflammation may not be a universal initiating factor for PCOS, but rather a pathological process activated within specific metabolic contexts. For instance, a study by Lindheim et al. did not identify intestinal barrier dysfunction or endotoxemia as primary drivers in their PCOS cohort (48); similarly, Lingaiah et al. observed no significant differences in markers such as zonulin and fatty acid-binding protein 2 (FABP2) between the PCOS group and BMI-matched controls, finding instead that these markers were more closely associated with BMI and insulin resistance (49). Kabakchieva evaluated the levels of the adipokine meteorin-like protein (Metrnl) and zonulin—a protein associated with intestinal permeability—in patients with PCOS to investigate their relationships with obesity, clinical manifestations, hormone levels, and metabolic parameters (50). The study found that while zonulin does not serve as a stable diagnostic marker for PCOS, there are links between gut barrier-related molecules and obesity, insulin resistance, and inflammatory status. Consequently, gut-derived inflammation likely exerts a pro-inflammatory effect primarily in patient subgroups exhibiting specific metabolic phenotypes, such as insulin resistance: gut microbiota dysbiosis and metabolic abnormalities synergistically compromise the intestinal barrier, facilitating the entry of LPS into the bloodstream and activating the TLR4/NF-κB pathway. This process further disrupts insulin signaling and ovarian steroidogenesis, creating a vicious cycle in which metabolic disturbances, inflammatory responses, and reproductive dysfunction reinforce one another. Notably, this inflammation-amplifying effect driven by microbiota dysbiosis often intertwines with the aforementioned imbalance in SCFA metabolism; aberrant fermentation under conditions of energy excess not only alters the SCFA profile but also impairs intestinal barrier function, collectively forming the complex pathological basis of PCOS-related gut microecological dysregulation.

#### 2.3.4 Enterohepatic circulation of BAs and sex hormones

In addition to participating in SCFA metabolism and the regulation of gut-derived inflammation, gut microbiota may influence PCOS-associated endocrine disorders by altering BA metabolic profiles. BAs are synthesized in the liver from cholesterol and enter the intestine via bile after conjugation with glycine or taurine; meanwhile, bacterial enzymes such as bile salt hydrolase and 7α-dehydroxylase convert primary BAs into various secondary BAs, including deoxycholic acid and lithocholic acid (51). Alterations in the structure or function of the gut microbiota can affect BA synthesis, conversion, and enterohepatic circulation, thereby modulating the activation status of BA-sensing receptors such as the farnesoid X receptor (FXR) and G protein-coupled BA receptor 1 (GPBAR1) (52). Thus, BAs serve not only as classic nutrients facilitating lipid digestion and absorption but also as important signaling molecules involved in glucose and lipid metabolism, gut hormone secretion, and reproductive endocrine regulation.

Extensive research indicates that patients with PCOS exhibit abnormal BA profiles, characterized primarily by changes in BA composition and synthesis pathways. Zhang et al. found elevated levels of circulating glycine- and taurine-conjugated primary BAs in PCOS patients, with these conjugated BAs showing a positive correlation with total testosterone and androstenedione levels (53). In non-obese women with PCOS, Zhu et al. observed an increased proportion of chenodeoxycholic acid (CDCA) and a decreased cholic acid (CA)/CDCA ratio—indirectly reflecting the depletion or downregulation of the hepatic enzyme cytochrome P450 family 8 subfamily B member 1 (CYP8B1) (54). The proportion of CDCA positively correlated with total testosterone and the free androgen index (FAI), suggesting that abnormal BA profiles may contribute to the hyperandrogenic state associated with PCOS. Yu et al. further observed elevated serum levels of CDCA, lithocholic acid, deoxycholic acid, and taurochenodeoxycholic acid, alongside reduced levels of glycodeoxycholic acid in PCOS patients; these findings confirm that BA abnormalities in PCOS encompass metabolic pathways involving both primary BA synthesis and the remodeling of conjugated BAs (55). Collectively, these findings indicate that abnormal BA metabolism is not merely secondary to obesity or insulin resistance but is linked to the hyperandrogenic phenotype of PCOS itself. Alterations in BAs may also extend to the local ovarian microenvironment. Yang et al. detected elevated levels of glycocholic acid, glycochenodeoxycholic acid, and CDCA glucuronide in the follicular fluid of PCOS patients, with overall levels of primary and conjugated BAs exceeding those of the control group (56). Notably, glycochenodeoxycholic acid levels positively correlated with serum follicle-stimulating hormone (FSH) and LH levels, while CDCA conjugates were associated with an increased antral follicle count. Although these findings do not establish a direct causal relationship between bile acids and follicular arrest, bile acids may still contribute to ovulatory dysfunction after entering the follicular microenvironment. Their effects may involve changes in granulosa cell function, follicular maturation, or local receptor signaling within the ovary.

The gut microbiota may also influence endocrine function by regulating the enterohepatic circulation of estrogens. After being conjugated through glucuronidation or sulfation in the liver, estrogens are secreted into the intestine through bile. β-Glucuronidase produced by certain intestinal bacteria can deconjugate these compounds, allowing the active estrogens to be reabsorbed into the circulation. Estrogens that remain conjugated are more likely to be excreted in the feces (57). Consequently, alterations in microbiota composition and associated enzymatic activities can influence circulating levels of active estrogens and the ratios of various estrogen metabolites. However, clinical evidence regarding the direct involvement of estrogens in the pathogenesis of PCOS remains limited; furthermore, a bidirectional relationship likely exists between the microbiota and sex hormones, rather than the microbiota simply driving hormonal abnormalities. Conversely, the hyperandrogenic environment characteristic of PCOS may reshape the gut ecosystem, creating an interdependent cycle involving microbial metabolism, BA signaling, and sex hormone regulation.

**Figure 1:**
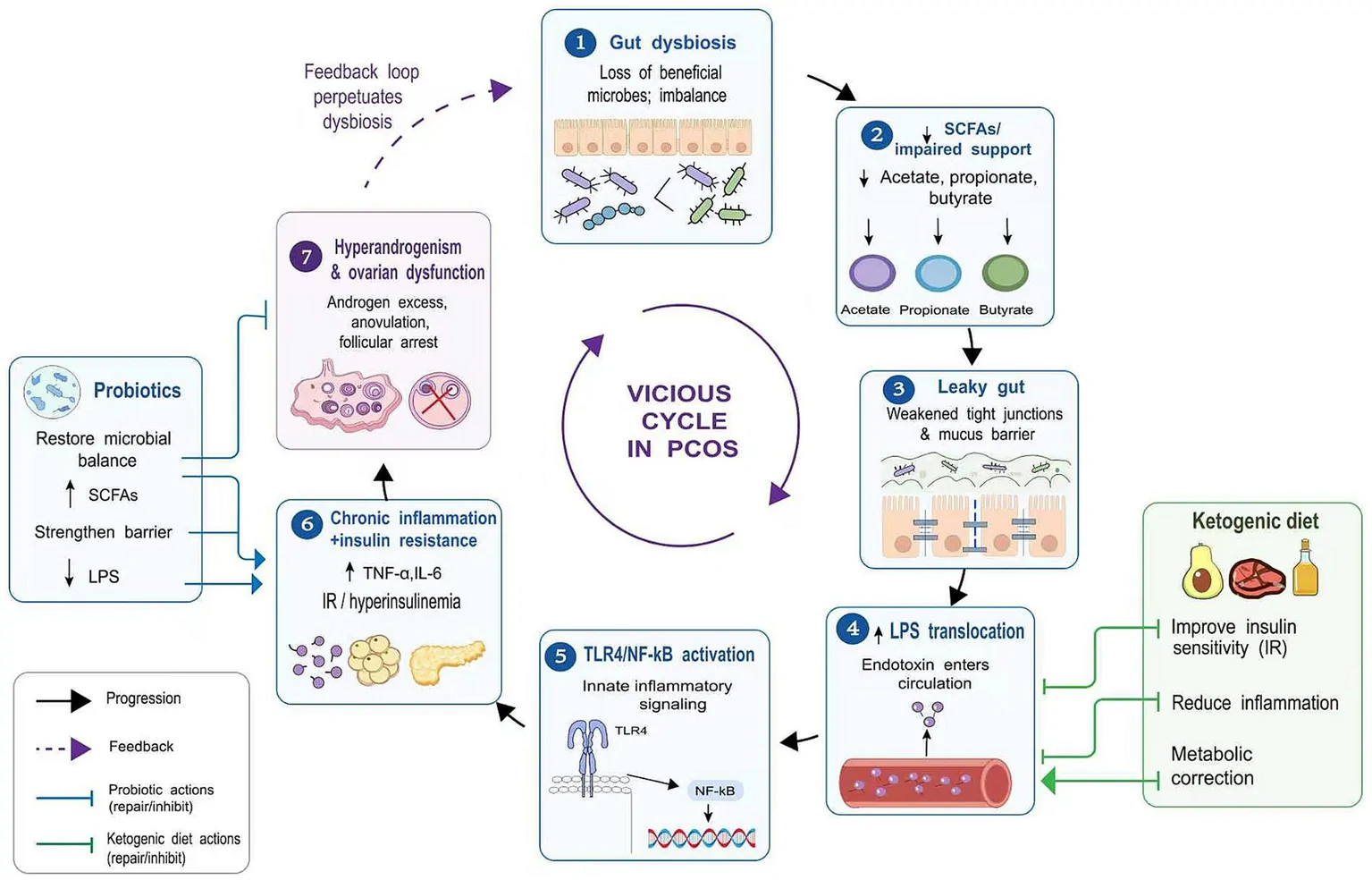
Proposed gut microbiota–metabolic–inflammatory vicious cycle in PCOS and potential intervention points of probiotics and KD. Gut dysbiosis in PCOS is characterized by the loss of beneficial microbes and microbial imbalance, leading to reduced production of SCFAs, including acetate, propionate, and butyrate. Decreased SCFA-mediated support weakens intestinal tight junctions and mucus barrier integrity, resulting in increased gut permeability and LPS translocation into the circulation. Circulating LPS activates innate immune signaling through the TLR4/NF-κB pathway, thereby promoting chronic low-grade inflammation, insulin resistance, and compensatory hyperinsulinemia. These metabolic and inflammatory disturbances further contribute to hyperandrogenism, anovulation, follicular arrest, and ovarian dysfunction, forming a self-perpetuating feedback loop. Probiotics may help restore microbial balance, enhance SCFA production, strengthen intestinal barrier function, and reduce LPS leakage, whereas KD may improve insulin sensitivity, attenuate inflammation, and promote metabolic correction. PCOS, polycystic ovary syndrome; SCFAs, short-chain fatty acids; LPS, lipopolysaccharide; TLR4, toll-like receptor 4; NF-κB, nuclear factor kappa B; IR, insulin resistance; TNF-α, tumor necrosis factor-alpha; IL-6, interleukin-6.

In summary, the gut microbiota shapes the complex metabolic-reproductive phenotype of PCOS through multiple mechanisms, including dysregulated SCFA metabolism, gut-derived low-grade inflammation, and aberrant BA signaling. Despite heterogeneity in microbiota characteristics across studies, existing evidence indicates that gut dysbiosis is triggered within specific metabolic contexts—such as insulin resistance and hyperandrogenism—and subsequently exacerbates endocrine disturbances via the gut–liver–ovarian axis. Notably, the regulation between the microbiota and the host is bidirectional; the hyperandrogenic environment characteristic of PCOS may, in turn, reshape the gut microecology. Future studies should further examine how microbial metabolism interacts with the genetic, metabolic, and hormonal characteristics of the host. A better understanding of these relationships may help develop more targeted microbiota-based interventions for different PCOS subtypes (Figure 1).

### 2.4 Gut–immune microenvironment perturbations in PCOS: overlapping mechanisms with gastrointestinal disorders

PCOS is increasingly recognized as a systemic endocrine–metabolic disorder in which gut microbial dysbiosis, impaired intestinal barrier integrity, and chronic low-grade inflammation interact with insulin resistance and hyperandrogenism. These alterations partially overlap with mechanisms observed in immune-related gastrointestinal disorders, particularly epithelial barrier disruption, translocation of microbial products, and activation of innate immune signaling. Viewing PCOS within this gut–immune framework may therefore help explain how intestinal perturbations contribute to extraintestinal metabolic and reproductive manifestations (58).

#### 2.4.1 Intestinal barrier dysfunction as a common denominator

The intestinal epithelial barrier is a critical interface between the gut microbiota and the host immune system. In PCOS, gut microbial dysbiosis and altered microbial metabolite production may impair mucus-layer function and tight-junction integrity, thereby increasing intestinal permeability. This process facilitates the translocation of microbial products, particularly lipopolysaccharide (LPS), into the systemic circulation, where they activate inflammatory pathways such as TLR4/MyD88/NF-κB signaling. The resulting chronic low-grade inflammation may further aggravate insulin resistance, hyperinsulinemia, androgen excess, and ovarian dysfunction (59).

Similar barrier-centered mechanisms are well established in immune-related gastrointestinal disorders, in which epithelial injury and persistent exposure to microbial antigens promote mucosal immune activation. However, intestinal barrier impairment may not be a universal feature of PCOS and appears to be more evident in metabolically compromised subgroups, particularly patients with obesity or insulin resistance. Therefore, intestinal barrier dysfunction should be considered a context-dependent mechanistic link connecting gut dysbiosis, immune activation, and metabolic–reproductive abnormalities in PCOS (60).

#### 2.4.2 PCOS as a systemic manifestation of gut-centric immune dysregulation

Viewing PCOS as a systemic disorder involving both reproductive endocrine dysfunction and alterations in the gut–immune environment may help explain its shared mechanisms with immune-related gastrointestinal diseases. In both settings, gut microbial dysbiosis and impaired epithelial barrier function may increase host exposure to microbial antigens and promote sustained activation of innate immune pathways. However, the magnitude, tissue localization, and clinical consequences of these alterations differ substantially between PCOS and primary gastrointestinal inflammatory diseases.

In inflammatory bowel disease, disruption of epithelial tight junctions, mucus-layer integrity, and mucosal immune tolerance represents a central component of intestinal pathology. Altered expression of tight-junction proteins, including claudins and occludin, together with changes in intestinal permeability-related molecules such as zonulin, contributes to increased exposure of mucosal immune cells to luminal microbial products. By contrast, intestinal barrier abnormalities in PCOS appear to be less pronounced and more heterogeneous. Increased intestinal permeability and elevated circulating markers of microbial translocation have been reported particularly in patients with obesity, insulin resistance, or other metabolic abnormalities (61)

Once the intestinal barrier is impaired, microbial products such as LPS can enter the circulation and activate the TLR4/MyD88/NF-κB pathway. This may increase the release of pro-inflammatory cytokines and contribute to persistent low-grade systemic inflammation. In PCOS, this inflammatory response may further impair insulin signaling, enhance compensatory hyperinsulinemia, and stimulate ovarian androgen production, linking intestinal perturbations to reproductive–endocrine dysfunction. Thus, rather than indicating that PCOS and inflammatory gastrointestinal disorders share an identical pathogenesis, current evidence suggests that they partially converge on a barrier–microbiota–innate immunity axis. This shared pathway may help explain why nutritional and microbiota-based interventions could benefit some patients with PCOS, although the response may differ among PCOS phenotypes.

## 3 KD improves PCOS: from animal models to clinical evidence

### 3.1 Overview of the KD

The KD is a dietary pattern characterized by high fat, moderate protein, and very low carbohydrate intake. Based on the degree of carbohydrate restriction and caloric control strategies, the KDs currently used in clinical practice are primarily categorized as follows:

1. Classic Ketogenic Diet (CKD): A fat-to-(protein + carbohydrate) ratio of 4:1 (by weight), with approximately 80–90% of calories derived from fat and carbohydrate intake typically <20 g/day; primarily used for refractory epilepsy (62).
2. Very-Low-Calorie Ketogenic Diet (VLCKD): Daily caloric intake <800 kcal, with 30–50 g/day of carbohydrates, 1.2–1.5 g/kg of protein, and 20–40 g/day of fat; this diet comprises three phases—active, re-education, and maintenance—and has received a weak recommendation from the Italian Society of Endocrinology (SIE) for obesity associated with PCOS (63).
3. Low-Calorie Ketogenic Diet (LCKD): Daily caloric intake >800 kcal but below total energy expenditure, with 30–50 g/day of carbohydrates; this is the most commonly used type in clinical studies regarding PCOS.
4. Modified Atkins Diet (MAD): A fat-to-(protein + carbohydrate) ratio between 1:1 and 2:1, with carbohydrate intake restricted to 10–20 g/day and no restrictions on protein or total calories, resulting in relatively high adherence (64).

When carbohydrate reduction leads to the depletion of hepatic glycogen, the body shifts to fat breakdown, producing ketone bodies (such as β-HB and AcAc) in the liver to supply energy. Beyond serving as an energy source, β-hydroxybutyrate (β-HB) also appears to have important signaling functions. It can inhibit histone deacetylases and influence the expression of genes involved in metabolism and inflammation. β-HB has also been reported to suppress NLRP3 inflammasome activation, affect oxidative stress responses, and regulate pathways involved in cellular energy sensing. These findings suggest that the effects of a ketogenic diet are not limited to carbohydrate restriction, but also involve broader metabolic, inflammatory, and cellular adaptations. Patients with PCOS commonly exhibit metabolic abnormalities such as obesity, hyperinsulinemia, insulin resistance, and chronic low-grade inflammation—pathological changes that are key targets directly addressed by the KD. Two meta-analyses from 2025 indicate that the KD significantly improves body weight, waist circumference, fasting blood glucose, fasting insulin, HOMA-IR, and androgen levels in patients with PCOS, suggesting it may serve as an effective nutritional intervention strategy within the comprehensive management of the condition (65).

The effects of ketogenic diets on the gut microbiota may not be attributable solely to ketone body production. Differences in dietary fiber intake, total energy restriction, and the proportion of saturated and unsaturated fatty acids may independently influence microbial composition, intestinal barrier function, and inflammatory responses. These dietary differences should therefore be considered when comparing findings across studies.

### 3.2 KD improves metabolic abnormalities in PCOS

The KD can effectively promote weight loss through mechanisms that include lowering insulin levels, enhancing fat mobilization, increasing fatty acid β-oxidation, and boosting satiety. Insulin resistance is considered a core pathological feature of PCOS. Studies indicate that approximately 70 to 80% of PCOS patients exhibit varying degrees of insulin resistance; even among patients with normal body weight, insulin sensitivity is significantly lower than in healthy women. Persistent insulin resistance triggers a compensatory hypersecretion of insulin. Excess insulin not only promotes fat accumulation and metabolic dysfunction but also acts directly on ovarian theca cells to enhance LH-induced androgen synthesis, thereby exacerbating hyperandrogenism and ovulatory dysfunction (66). Consequently, alleviating insulin resistance has become a key therapeutic goal for PCOS.

The KD ameliorates PCOS-related metabolic abnormalities by reducing glucose load and the demand for insulin secretion. Traditional high-carbohydrate diets cause rapid postprandial blood glucose spikes, stimulating pancreatic β-cells to secrete large amounts of insulin. In contrast, the KD strictly limits carbohydrate intake, significantly dampening postprandial blood glucose fluctuations and curbing insulin secretion at the source. As insulin levels decline, the receptor desensitization caused by prolonged exposure to high insulin concentrations is alleviated, allowing tissue insulin sensitivity to gradually recover (67). Furthermore, a low-insulin state relieves the inhibition of hormone-sensitive lipase (HSL), promoting the breakdown of adipose tissue and providing the body with fatty acids as an energy source, thereby further intensifying fat oxidation.

At the molecular level, the KD modulates multiple signaling pathways closely linked to energy metabolism. Research suggests that the ketogenic state enhances the activity of AMP-activated protein kinase (AMPK), a crucial cellular energy sensor. Upon activation, AMPK promotes the translocation of glucose transporter 4 (GLUT4) to the cell membrane, thereby increasing glucose uptake in skeletal muscle and adipose tissue; simultaneously, it inhibits fatty acid and cholesterol synthesis while promoting fatty acid oxidation (68). Furthermore, AMPK negatively regulates the mechanistic target of rapamycin (mTOR) signaling pathway, thereby alleviating insulin resistance and inflammatory responses in adipose tissue (69). These combined effects contribute to improving the metabolic status of patients with PCOS.

Obesity is one of the most common comorbidities associated with PCOS. Epidemiological data indicate that over 50% of patients with PCOS are overweight or obese. Excessive visceral fat accumulation not only exacerbates insulin resistance but also promotes disease progression through the secretion of inflammatory factors and adipokines. Ketone bodies possess appetite-suppressing properties and can reduce energy intake by influencing the hypothalamic appetite-regulating centers. Additionally, a relatively higher proportion of protein intake helps enhance satiety. Mavropoulos et al. conducted one of the early clinical studies on the KD in the context of PCOS, implementing a low-carbohydrate ketogenic diet for 24 weeks (70). The results showed that participants who completed the study experienced an average weight loss of 12%, significant reductions in fasting insulin and free testosterone levels, and, in some cases, the restoration of normal menstrual cycles. This study was the first to suggest that a KDt could not only facilitate weight loss but also improve endocrine abnormalities associated with PCOS. These findings were also supported by later clinical studies. A recent RCT comparing a KD with a moderate-carbohydrate diet in overweight or obese women with PCOS found that the KD group outperformed the control group in reducing body weight, BMI, waist circumference, and body fat percentage; significant reductions in fasting blood glucose, insulin levels, and HOMA-IR were also observed (71). Moreover, the KD intervention led to decreased total testosterone levels and improved menstrual regularity, demonstrating positive effects on both metabolic abnormalities and hyperandrogenism. A study on the VLCKD observed similar results, finding that the induction of nutritional ketosis led to significant reductions in body weight and visceral fat, as well as fasting insulin and HOMA-IR, alongside increased sex hormone-binding globulin (SHBG) and a decreased FAI (72). These results suggest that improved insulin sensitivity may be a key mechanism by which the KD alleviates hyperandrogenemia. Furthermore, most intervention trials have shown that adopting a KD leads to improvements in weight management, glucose and lipid metabolism, and androgen levels, with some patients also experiencing a restoration of ovulatory function (73). These effects are generally observed after 8–12 weeks of intervention, while longer treatment periods may lead to greater improvements in metabolic outcomes.

Current evidence suggests that the ketogenic diet may improve metabolic disturbances in women with PCOS by lowering dietary carbohydrate intake, improving insulin sensitivity, increasing fat oxidation, and regulating glucose and lipid metabolism. These changes may support weight management and may also help reduce androgen excess and improve reproductive function. In addition, the ketogenic diet may affect inflammation, oxidative stress, and the gut microbiota, although these effects and their clinical importance require further investigation.

### 3.3 KD improves reproductive-endocrine function in PCOS by modulating gut microbiota and inflammatory responses

The “gut microbiota–metabolite–immune regulation” network is considered a crucial theoretical framework for elucidating the mechanisms by which the KD ameliorates PCOS. Recent studies have shown that the KD significantly alters gut microbiota composition, with a mode of action distinct from that of a traditional high-fat diet. Research by Ang et al., involving both human and animal models, demonstrated that the KD induces rapid remodeling of the gut microbiota and significantly reduces the levels of pro-inflammatory Th17 cells in the gut (74). Further studies suggest that these microbial changes are not explained solely by the higher fat content of the diet, but may also be related to increased ketone body production. In particular, β-HB may alter the abundance of specific gut bacteria and, in turn, affect host metabolism and immune function.

The effects of the ketogenic diet on the gut microbiota also appear to be taxon-specific. During ketosis, the abundance of several bacteria associated with metabolic health, including *Akkermansia* and *Parabacteroides*, has been reported to increase. These bacteria help maintain intestinal barrier integrity, promote mucus layer metabolism, and suppress inflammatory responses, thereby fostering conditions conducive to host metabolic homeostasis. However, studies have clearly demonstrated that the β-HB produced during the KD directly inhibits the growth of *Bifidobacterium*, representing the most significant “negative effect” of the diet on the microbiota (74). Given that *Bifidobacterium* is a key probiotic genus for maintaining intestinal homeostasis and producing SCFAs, its depletion may lead to reduced microbial diversity and compromised microecological stability. The ketogenic diet may therefore have mixed effects on the gut microbiota. Although it can improve metabolic outcomes, it may also disrupt certain beneficial microbial populations.

Changes in microbial composition are often accompanied by shifts in microbial metabolites. Short-chain fatty acids and bile acids are among the main metabolites involved in communication between the gut microbiota and host metabolism. In a mouse model, Yang et al. found that SCFAs—such as acetate, propionate, and butyrate—promote the secretion of interleukin-22 (IL-22) by innate lymphoid cells (ILCs) and CD4^+^ T cells. Furthermore, these SCFAs enhance intestinal mucosal immune function through the activation of the GPR41 signaling pathway and the inhibition of HDACs (75). IL-22 not only maintains intestinal epithelial integrity but also promotes the expression of antimicrobial peptides and mucus secretion, thereby fostering an intestinal microenvironment conducive to host metabolic health. Beyond SCFAs, a KD significantly alters BA composition and enterohepatic circulation, subsequently influencing key signaling pathways such as FXR and Takeda G protein-coupled receptor 5 (TGR5). FXR and TGR5 are involved not only in the regulation of glucose and lipid metabolism but also in promoting GLP-1 secretion and modulating energy homeostasis and inflammatory responses (76). Consequently, KD-induced remodeling of the gut microbiota may further ameliorate metabolic abnormalities in patients with PCOS by altering SCFA and BA metabolism.

Changes in metabolites further influence the body’s immune-inflammatory status. In recent years, chronic low-grade inflammation has been identified as a crucial link between gut microbiota dysbiosis and the pathogenesis of PCOS. Patients with PCOS typically exhibit elevated serum levels of inflammatory factors such as TNF-α, IL-6 (77), and C-reactive protein (CRP); these inflammatory mediators not only exacerbate insulin resistance but also directly impair ovarian granulosa cell function and affect follicular development. Research indicates that -βHB—the primary ketone body produced during a KD—exerts significant immunomodulatory effects in addition to serving as an energy substrate; it inhibits the expansion of pro-inflammatory Th17 cells and reduces inflammation in both the gut and peripheral tissues. Animal studies have further demonstrated that a KD can alleviate inflammation by modulating gut microbiota composition, suppressing pro-inflammatory immune responses (such as IL-17 signaling), and improving intestinal barrier function (78).

Concurrently, a KD improves intestinal barrier function. Compromised intestinal barriers allow bacterial products, such as LPS, to enter the circulatory system, triggering metabolic endotoxemia and sustaining inflammatory responses (79). A higher abundance of Akkermansia has been associated with a thicker intestinal mucus layer and increased expression of tight junction proteins, which may help reduce intestinal permeability and inflammatory activity (80). Because impaired intestinal barrier function and chronic low-grade inflammation are common in PCOS, the ketogenic diet may help reduce systemic inflammation by improving barrier integrity and limiting the entry of LPS into the circulation.

Improvements in inflammatory status are reflected in the restoration of reproductive endocrine function. Both chronic inflammation and hyperinsulinemia promote androgen synthesis by ovarian theca cells and inhibit hepatic production of SHBG, resulting in elevated free testosterone levels. As the KD induces gut microbiota remodeling, modulates microbial metabolites, and reduces inflammation, the body’s insulin sensitivity gradually improves; this alleviates the hyperandrogenic state and facilitates the restoration of ovulatory function. Studies have observed that KD intervention leads to decreased total testosterone levels and an improved LH/FSH ratio, alongside increased menstrual regularity (20). These findings suggest that the underlying mechanisms extend beyond weight loss alone, involving the systemic regulation of gut microbiota and immunometabolic networks.

**Figure 2:**
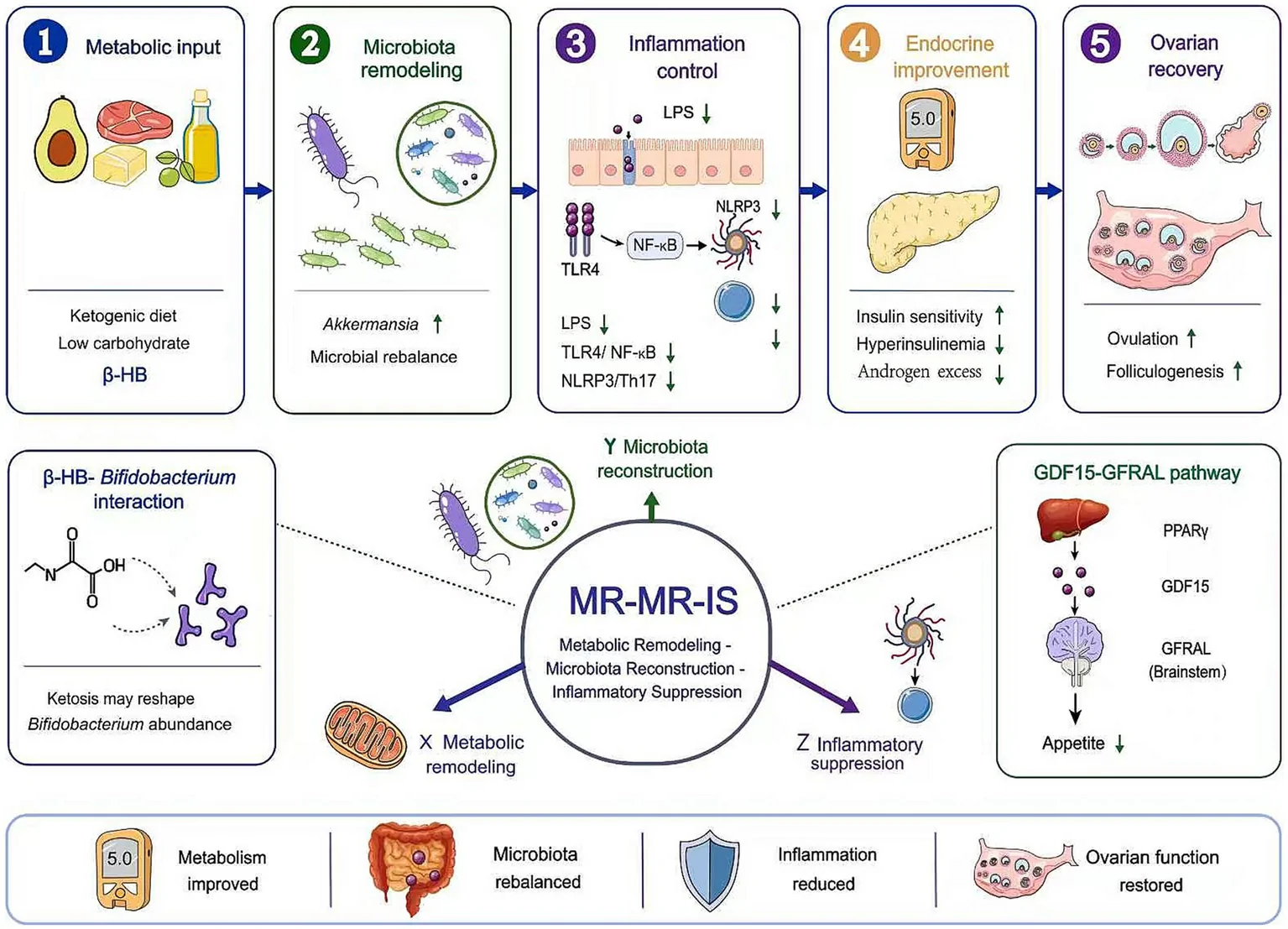
KD-mediated metabolic remodeling–microbiota reconstruction–inflammatory suppression axis in PCOS. A KD, characterized by low carbohydrate intake and increased ketone body production, may initiate metabolic remodeling through β-HB and related metabolic signals. This metabolic shift may reshape the gut microbiota, including increased *Akkermansia* and altered *Bifidobacterium* abundance, thereby contributing to microbial rebalance. Improved gut microbial homeostasis may reduce LPS leakage and suppress inflammatory signaling through the TLR4/NF-κB and NLRP3/Th17 pathways. The attenuation of chronic inflammation further improves insulin sensitivity, reduces hyperinsulinemia and androgen excess, and ultimately promotes ovulation, folliculogenesis, and ovarian functional recovery. Additional regulatory mechanisms, including β-HB–*Bifidobacterium* interactions and the GDF15–GFRAL appetite-regulatory pathway, may further link KD-induced metabolic changes with microbiota reconstruction, inflammatory suppression, and endocrine improvement in PCOS. PCOS, polycystic ovary syndrome; KD, ketogenic diet; β-HB, β-hydroxybutyrate; LPS, lipopolysaccharide; TLR4, toll-like receptor 4; NF-κB, nuclear factor kappa B; NLRP3, NOD-like receptor family pyrin domain-containing 3; Th17, T helper 17 cell; GDF15, growth differentiation factor 15; GFRAL, glial cell line-derived neurotrophic factor family receptor alpha-like; PPARγ, peroxisome proliferator-activated receptor gamma; MR-MR-IS, metabolic remodeling–microbiota reconstruction–inflammatory suppression.

Overall, the ketogenic diet may improve PCOS through several related mechanisms, including changes in gut microbial composition, alterations in short-chain fatty acid and bile acid metabolism, improved intestinal barrier function, and reduced chronic inflammation. These effects may contribute not only to weight loss and better insulin sensitivity, but also to improvements in androgen levels and reproductive function (Figure 2 and Table 1).

**Table 1:** Summary of clinical trials on the KD for improving PCOS.

Study (Ref.)	Sample size	KD type	Duration	BMI changes	HOMA-IR	Testosterone	Menstrual-related outcomes
Paoli et al. 2020 (20)	14 overweight women with PCOS	Low-calorie Mediterranean ketogenic diet protocol (KEMEPHY)	12 weeks	BMI decreased by approximately 3.35 kg/m^2^; body weight decreased by approximately 9.43 kg	Significantly improved; 2.85 ± 0.15 before treatment vs. 2.32 ± 0.13 after treatment	Total testosterone decreased from 47.43 ng/dL before treatment to 40.71 ng/dL after treatment; free testosterone decreased from 0.96 pg./mL to 0.56 pg./mL	Estradiol, progesterone, and sex hormone-binding globulin (SHBG) levels increased
Rossetti et al. 2024 (21)	18 patients with PCOS enrolled; 12 completed the study	Daily carbohydrate intake <50 g; daily protein intake 1.3–1.4 g/kg	KD for 45 days, followed by 45 days of gradual carbohydrate reintroduction; follow-up to 6 months	Body weight/body fat decreased; mean body weight decreased by approximately 2 kg	Trend toward improvement (*p* = 0.076)	Testosterone and hirsutism improved	Menstrual cycles improved significantly (*p* = 0.012); ovarian volume was significantly reduced, indicating improvement in ovarian morphology and polycystic features (*p* = 0.029)
Mavropoulos et al. 2005 (70)	11 enrolled; 5 completed	Low-carbohydrate ketogenic diet; carbohydrates ≤20 g/day	24 weeks	Mean body weight decreased by approximately 12% (BMI decreased along with body weight)	Fasting insulin decreased by approximately 54%	Free testosterone decreased by approximately 22%	Luteinizing hormone/follicle-stimulating hormone ratio decreased by 36%; 2 pregnancies were reported
Sharifi et al. 2024 (71)	46 enrolled; 19 completed in the KD group and 21 in the control group	KD: 10% carbohydrate, 20% protein, and 70% fat, with daily carbohydrate intake <30 g; control diet: 40% carbohydrate, 20% protein, and 40% fat	8 weeks	Both groups showed decreases; the reduction was greater in the KD group (KD: 5.64 kg; control: 4.35 kg)	Fasting insulin and HOMA-IR decreased more markedly in the KD group	Androgen indicators, including free testosterone and DHEAS, decreased	Not systematically reported as a primary outcome
Li, J et al. 2021 (73)	20 enrolled; 18 completed	KD: 5–10% carbohydrate, 18–27% protein, and 70–75% fat, with daily carbohydrate intake <50 g	12 weeks	Body weight decreased from 87.19 to 75.41; body fat percentage decreased from 35.38 to 28.57	Indices of glucose metabolism/insulin resistance improved	Sex hormone indicators improved	Menstrual cycles shortened

KD, ketogenic diet; PCOS, polycystic ovary syndrome; BMI, body mass index; HOMA-IR, homeostatic model assessment of insulin resistance; DHEAS, dehydroepiandrosterone sulfate.

## 4 Probiotic intervention for PCOS

### 4.1 Theoretical basis for probiotic intervention in PCOS

Probiotics, prebiotics, synbiotics, postbiotics, and FMT should not be treated as interchangeable interventions. Probiotics deliver live microorganisms, prebiotics provide selectively utilized substrates, synbiotics combine microbial and substrate components, postbiotics contain inactivated microorganisms or microbial products, and FMT transfers a complex microbial community. Their mechanisms, safety profiles, and levels of clinical evidence should therefore be evaluated separately. In patients with PCOS, gut microbiota dysbiosis is closely linked to pathological processes such as insulin resistance, chronic low-grade inflammation, abnormal lipid metabolism, and hyperandrogenism. Existing research suggests that PCOS is not merely a localized ovarian disorder but a systemic disease involving the interplay of gut microbiota, metabolic inflammation, and reproductive endocrine function. Consequently, interventions targeting the gut microbiota have emerged as a focal point of research in the comprehensive management of PCOS.

Probiotics—defined as live microorganisms that confer health benefits to the host when administered in adequate amounts—represent a key strategy for modulating the gut microbiota; common genera include *Lactobacillus* and *Bifidobacterium* (22). Unlike conventional drug treatments, which mainly address symptoms such as ovulatory dysfunction, androgen excess, and insulin resistance, probiotics act by modifying the gut microbiota. Changes in microbial composition and function may, in turn, influence the metabolic and endocrine disturbances associated with PCOS.

Probiotics may help improve PCOS by supporting the growth of beneficial gut bacteria and limiting bacteria that contribute to inflammation, thereby promoting a healthier balance in the gut microbiota. Probiotics may also strengthen the intestinal mucosal barrier and reduce the passage of gut-derived endotoxins, including LPS, into the circulation. This may help reduce the chronic low-grade inflammation commonly seen in women with PCOS (24). As inflammation decreases, insulin signaling may improve, which can help alleviate insulin resistance. Better insulin sensitivity may then reduce the effect of compensatory hyperinsulinemia on ovarian androgen production and support the recovery of SHBG levels, thereby contributing to an improvement in hyperandrogenism.

Probiotics may also act by altering the metabolites produced by the gut microbiota. Some probiotic strains can increase the production of short-chain fatty acids, particularly acetate, propionate, and butyrate. SCFAs not only provide energy to intestinal epithelial cells and maintain intestinal barrier integrity but also play roles in glucose and lipid metabolism, incretin secretion, and immune regulation. Studies have shown that *Bifidobacterium animalis* subsp. *lactis* V9 may influence sex hormone secretion in women with PCOS by altering the gut microbiota. Improvements in SCFA levels and clinical indicators were more evident in patients in whom the strain successfully colonized the intestine (81). Probiotics may also affect bile acid metabolism and the production of intestinal immune factors, which could contribute to interactions among the gut microbiota, immune regulation, and ovarian function.

These mechanistic studies provide a rationale for clinical intervention, and recent RCTs have further validated the potential efficacy of probiotics in PCOS management. For instance, a randomized, double-blind, placebo-controlled study by Karamali et al. (82) demonstrated that continuous supplementation with multi-strain probiotics in women with PCOS led to reductions in total testosterone, modified Ferriman-Gallwey scores, high-sensitivity C-reactive protein (hs-CRP), and markers of oxidative stress, alongside increases in SHBG and total antioxidant capacity; this suggests that probiotics may simultaneously modulate androgen levels, inflammatory responses, and oxidative stress status. Darvishi et al. also reported that synbiotic supplementation in women with PCOS improved several metabolic parameters, including fasting blood glucose, insulin levels, HOMA-IR, HDL cholesterol, and obesity-related measures (83). Although these studies were generally small and differed in the probiotic strains, doses, and treatment periods used, the available evidence suggests that probiotics may be a useful adjunct to conventional treatment and dietary management in women with PCOS.

Importantly, probiotic effects are strain-specific, and findings obtained with one strain should not be generalized to other strains within the same species or genus, because colonization capacity, metabolite production, bile acid transformation, and immunomodulatory activity may differ. Evidence from single-strain interventions, multi-strain formulations, and synbiotic preparations should also be interpreted separately, as the effects of individual strains cannot be isolated in multi-strain products, while prebiotic components in synbiotics may independently influence clinical outcomes.

### 4.2 Mechanisms by which probiotics improve metabolic, inflammatory, and endocrine abnormalities in PCOS

The potential effects of probiotics on PCOS are characterized by a multi-target nature; rather than simply improving a single hormonal marker, they comprehensively influence the metabolic abnormalities and reproductive-endocrine disorders associated with PCOS by reshaping gut microbiota composition, modulating the production of microbial metabolites, inhibiting chronic low-grade inflammation, and optimizing the synergistic function of metabolic-endocrine axes. Existing evidence suggests that changes in the gut environment may influence systemic metabolic and endocrine function through proposed mechanistic frameworks such as the gut–ovary axis and the gut–hypothalamus–pituitary axis. However, these pathways have not been fully established in women with PCOS. Based on this, the mechanisms by which probiotics affect PCOS can be summarized in the following key aspects.

First, probiotics can participate in the pathological regulation of PCOS by reshaping gut microbiota composition. Evidence from animal studies shows that in rat models of PCOS, interventions involving healthy FMT or exogenous *Lactobacillus* administration not only restore disrupted gut microecology but also alleviate estrous cycle irregularities and ovarian polycystic-like changes (84). Changes in the gut microbiota have been associated with higher levels of Lactobacillus and *Clostridium* and a lower abundance of *Prevotella*, together with reduced activity of androgen synthesis pathways. This suggests that shifts in the gut microbial community may be related to changes in sex hormone levels. Similar findings have been reported in letrozole-induced rat models of PCOS. Treatment with *Lactiplantibacillus plantarum* HL2 and *Bifidobacterium longum* HB3 improved gut microbial imbalance, increased colonic SCFA levels, and helped normalize LH, FSH, and testosterone levels (85). In a DHT-induced mouse model, supplementation with the probiotic strain BL21 also improved metabolic abnormalities, systemic inflammation, insulin sensitivity, and sex hormone profiles. These findings further suggest that changes in the gut microbiota and its metabolites may contribute to the metabolic and hormonal disturbances associated with PCOS.

Secondly, probiotics influence the onset and progression of PCOS not only by altering gut microbiota composition but also by regulating microbial metabolites and intestinal immune pathways. In a cohort study of PCOS patients combining 16S rRNA sequencing with clinical metabolic analysis, Liu et al. found that alterations in gut microbiota composition were closely associated with fasting insulin levels, HOMA-IR, and androgen levels; notably, a reduction in SCFA-producing bacteria correlated with the severity of metabolic disorders, suggesting that SCFA-related microbiota may participate in the pathological process of PCOS by regulating energy metabolism and endocrine balance (86). Meanwhile, using a PCOS-like mouse model and combining FMT with metabolomic analysis, Ho et al. discovered that gut microbiota from different sources could significantly reshape the host’s BA profile and energy metabolic state—manifesting as exacerbated insulin resistance, impaired ovarian function, and enhanced inflammatory signaling—whereas transplantation of healthy microbiota could partially reverse these abnormalities, further supporting the role of BA metabolism and related signaling networks in regulating the PCOS phenotype (87). Additionally, research by Wu et al. using a PCOS rat model demonstrated that *Akkermansia muciniphila* (PROBIO) could ameliorate reproductive dysfunction by promoting arginine biosynthesis, indicating that, alongside SCFAs and BAs, other microbial metabolic pathways may also play significant roles in the pathological regulation of PCOS (88).

Thirdly, probiotic intervention can alleviate PCOS-associated metabolic disorders by suppressing chronic low-grade inflammation. Evidence from RCTs indicates that probiotic supplementation over several weeks in women with PCOS leads to significant reductions in serum inflammatory markers—specifically IL-6, TNF-α, and hs-CRP—alongside improved insulin sensitivity; this suggests that probiotics can enhance glucose homeostasis by mitigating systemic inflammation (89). In a 12-week randomized clinical trial, Ji et al. compared probiotics, metformin, and their combination. Menstrual-cycle recovery and ovulation rates were highest in the combined probiotic–metformin group. BMI, fasting glucose, HOMA-IR, lipid profiles, AMH, testosterone, and FAI also improved after probiotic and/or metformin treatment (90). These findings suggest that probiotics may concurrently improve inflammatory, metabolic, and reproductive-endocrine outcomes. Furthermore, Jamilian et al. reported that combined probiotic and selenium supplementation was associated with reductions in hs-CRP, IL-6, and malondialdehyde (MDA) in women with PCOS, suggesting potential concurrent effects on inflammatory and oxidative-stress markers. However, the contribution of the probiotic component cannot be separated from that of selenium (91).

Fourth, probiotic intervention may improve certain reproductive-endocrine indicators in women with PCOS, potentially through indirect metabolic and inflammatory pathways. In a 12-week trial, Szydłowska et al. reported significant between-group decreases in TSH, androstenedione, and BMI and an increase in SHBG after multi-strain probiotic supplementation; testosterone and DHEAS did not show significant between-group improvement. These changes do not establish a direct ovarian effect of probiotics and may have occurred indirectly through improved insulin sensitivity, weight reduction, reduced inflammation, or increased hepatic SHBG synthesis (92). A six-month randomized, placebo-controlled trial by Chudzicka-Strugała et al. showed that, among overweight or obese women with PCOS, the addition of synbiotic supplementation to lifestyle intervention produced greater improvements in hyperandrogenism, lipid profiles, and markers of endotoxemia than lifestyle intervention alone (93). These findings suggest that long-term modulation of the gut microbiota may complement lifestyle management by improving both metabolic and endocrine abnormalities in PCOS. Furthermore, in a randomized, double-blind, placebo-controlled trial, Shirani et al. found that an 8-week intervention with *Lactobacillus helveticus* and *Bifidobacterium longum* increased SHBG, total antioxidant capacity, and superoxide dismutase and decreased FAI, CRP, and MDA. After adjustment, total testosterone did not differ significantly between groups, and acne, alopecia, and hirsutism showed no significant between-group improvement (94). Recent studies have also identified the FXR as a crucial signaling node linking microbial metabolism to the endocrine phenotype of PCOS. Yun et al. discovered that agmatine, produced by *Bacteroides vulgatus*, activates intestinal FXR and inhibits the secretion of GLP-1 from intestinal L-cells, subsequently inducing insulin resistance, elevated serum testosterone, and ovarian dysfunction; inhibiting agmatine production or administering GLP-1 receptor agonists alleviated these PCOS-like phenotypes (95). These findings suggest that gut bacteria may influence FXR signaling in more than one way. In addition to altering bile acid composition, they can produce metabolites that activate FXR and thereby affect insulin signaling and ovarian steroid hormone production. Because hyperinsulinemia can stimulate androgen production in ovarian theca cells and reduce hepatic SHBG synthesis, alterations in FXR signaling and gut hormone secretion may further worsen hyperandrogenism in women with PCOS.

Overall, probiotics may improve PCOS through several related mechanisms. By reshaping the gut microbiota, altering microbial metabolites, and reducing inflammation, they may help improve both metabolic and endocrine function. Increases in beneficial bacteria and changes in short-chain fatty acid, amino acid, and bile acid metabolism may support insulin sensitivity and hormone balance. Their anti-inflammatory effects may also help protect ovarian function from the effects of persistent low-grade inflammation. Crucially, through bidirectional regulation of the metabolic-endocrine axis, probiotics can facilitate a reduction in androgen levels, an increase in SHBG, and a decrease in BMI, thereby yielding synergistic improvements across both metabolic and reproductive-endocrine abnormalities. Although RCTs and animal studies have preliminarily demonstrated this potential, further systematic evaluation is required regarding strain specificity, intervention duration, dose–response relationships, and long-term efficacy. In the future, integrated with the concept of precision nutrition, probiotics hold promise as a key adjunct in the comprehensive management of PCOS—complementing lifestyle interventions and pharmacological treatments to provide patients with more holistic and sustainable support for metabolic and reproductive health.

### 4.3 Potential complementary effects of combined KD and probiotic intervention

KD and probiotic interventions in the management of PCOS focus on two key aspects—metabolic regulation and the gut microbiota—respectively; given the significant complementarity of their mechanisms of action, their combined application offers potential theoretical advantages.

#### 4.3.1 Coordination at the level of metabolic regulation

From a metabolic perspective, the ketogenic diet may improve insulin resistance by substantially reducing carbohydrate intake, limiting postprandial glucose excursions, and lowering circulating insulin levels (96). Recent clinical studies have reported reductions in HOMA-IR, fasting insulin, and free testosterone following ketogenic or very-low-carbohydrate diets, together with improvements in body weight and menstrual regularity. Probiotics may also support insulin sensitivity by altering gut microbial composition, increasing short-chain fatty acid production, and influencing incretin secretion. Results from multiple RCTs indicate that probiotic interventions can improve insulin resistance and inflammatory markers. Together with the reported metabolic effects of KD, these separate findings suggest potential metabolic complementarity; however, the combined intervention has not been directly tested in women with PCOS (97).

#### 4.3.2 Complementarity at the level of gut microecology

At the level of gut microecology, the KD induces a comprehensive remodeling of the gut microbiota—shifting it toward a state adapted to high-fat metabolism—by altering energy substrate availability and the BA metabolic environment. Recent studies in animal models of PCOS have shown that the ketogenic diet can alter gut microbial composition and metabolic profiles while improving insulin resistance. These microbial effects, however, may not always be beneficial. β-HB, for example, can suppress the growth of *Bifidobacterium* and reduce the abundance of bacteria involved in short-chain fatty acid production (98). Supplementation with selected probiotic strains may help restore microbial function and support SCFA production. It may also partly offset the reduction in *Bifidobacterium* associated with increased β-HB levels, suggesting that probiotics could complement the effects of the ketogenic diet at the level of the gut microbiota.

**Figure 3:**
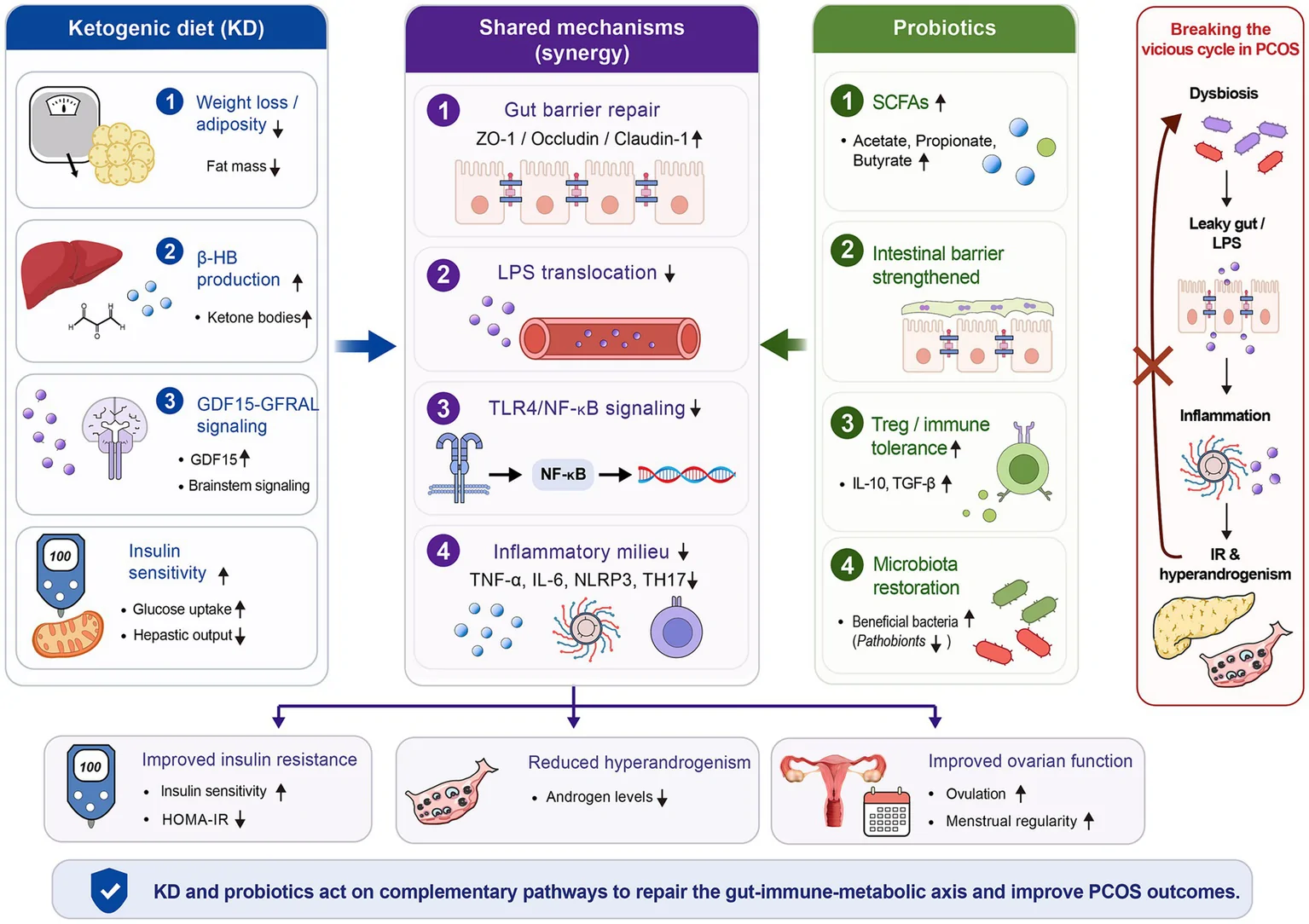
Complementary mechanisms of KD and probiotics in breaking the gut–immune–metabolic vicious cycle in PCOS. KD and probiotics may improve PCOS through complementary regulation of metabolic, microbial, intestinal barrier, and inflammatory pathways. KD contributes to weight loss and reduced adiposity, increases ketone body production, particularly β-HB, activates GDF15–GFRAL-mediated brainstem signaling, and improves insulin sensitivity by enhancing glucose uptake and reducing hepatic glucose output. Probiotics promote SCFA production, including acetate, propionate, and butyrate, strengthen intestinal barrier integrity, enhance Treg-mediated immune tolerance, and restore gut microbial balance by increasing beneficial bacteria and reducing pathobionts. These interventions converge on shared mechanisms, including gut barrier repair, decreased LPS translocation, suppression of TLR4/NF-κB signaling, and attenuation of inflammatory mediators such as TNF-α, IL-6, NLRP3, and Th17-related inflammation. Through these coordinated effects, KD and probiotics may reduce insulin resistance and hyperandrogenism, improve ovulation and menstrual regularity, and ultimately contribute to ovarian functional recovery in PCOS. PCOS, Polycystic ovary syndrome; KD, Ketogenic diet; β-HB, β-hydroxybutyrate; GDF15, Growth differentiation factor 15; GFRAL, Glial cell line-derived neurotrophic factor family receptor alpha-like; SCFA, Short-chain fatty acid; Treg, Regulatory T cell; LPS, Lipopolysaccharide; TLR4, Toll-like receptor 4; NF-κB, Nuclear factor kappa B; TNF-α, Tumor necrosis factor-alpha; IL-6, Interleukin-6; NLRP3, NOD-like receptor family pyrin domain-containing 3; Th17, T helper 17 cell; HOMA-IR, Homeostatic model assessment of insulin resistance; IR, Insulin resistance.

Regarding inflammation regulation, the KD primarily lowers systemic metabolic inflammation and reduces the expression of CRP and pro-inflammatory cytokines by improving insulin resistance and lipid metabolism. Probiotics, in contrast, act predominantly on gut-derived inflammatory pathways; they alleviate chronic inflammation caused by gut dysbiosis by enhancing intestinal mucosal barrier function, reducing LPS translocation, and inhibiting the activation of the TLR4/NF-κB signaling pathway. Recent research further demonstrates that probiotic or synbiotic interventions can significantly reduce the inflammatory burden associated with PCOS and improve metabolic status. By intervening at the levels of systemic metabolic inflammation and gut-derived endotoxin-induced inflammation respectively, the two approaches may have potentially complementary anti-inflammatory mechanisms, offering new perspectives for the comprehensive management of PCOS. However, no clinical study has directly demonstrated that combining a ketogenic diet with probiotics improves intestinal barrier integrity in women with PCOS; therefore, this proposed barrier-related benefit should be regarded as a mechanistic hypothesis rather than an established clinical effect (Figure 3).

### 4.4 Limitations and complementarity of individual interventions

Although both the KD and probiotic supplementation show potential benefits in the management of PCOS, applying either in isolation has limitations. As a strict dietary intervention, the KD effectively improves insulin resistance and weight-related metabolic markers in the short term; however, long-term adherence is often poor. However, very low carbohydrate intake also increases β-HB, which has been reported to suppress the growth of *Bifidobacterium*. This may reduce SCFA production and alter microbial diversity, potentially affecting the stability of the gut microbiota. Responses to the KD also vary considerably between individuals, which may partly explain why clinical outcomes are not consistent across different populations.

Probiotic interventions also have several limitations. Their effects depend largely on the strains and combinations used, and successful colonization is not always achieved. Even when supplemented bacteria increase initially, they may not remain in the gut after treatment, particularly when the intestinal environment does not support their long-term survival. These differences make it difficult to establish a standardized probiotic regimen for PCOS.

The KD and probiotics may therefore have complementary roles. By altering the intestinal metabolic environment and microbial community, the ketogenic diet may create conditions that influence the response to probiotic supplementation. Probiotics, particularly *Bifidobacterium* and butyrate-producing strains, may help replace bacteria reduced during ketosis, support microbial function, and partly offset the inhibitory effects of β-HB on beneficial bacteria.

### 4.5 Current status and future directions of combined interventions

No randomized controlled trial has directly evaluated the combined use of a KD and probiotics in women with PCOS. Current evidence is derived mainly from studies evaluating the two interventions separately or from related populations. Therefore, any potential complementary or additional benefits remain hypothetical and require clinical validation.

However, few studies have directly investigated how these two interventions may interact within the gut microbiota. It therefore remains uncertain whether their combined use produces additive or synergistic effects on short-chain fatty acid production, bile acid metabolism, and intestinal immune signaling. Furthermore, inter-individual variability in responses to dietary modifications and microbiota-targeted interventions—along with the underlying determinants of such variability—has not been fully elucidated. A systematic review and meta-analysis by Wang et al. demonstrated that, in obese populations, a VLCKD combined with probiotics or prebiotics improved gut microbiota diversity and metabolic parameters more effectively than a VLCKD alone; this provides indirect support for combined interventions in the context of PCOS. Nevertheless, dedicated RCTs are required to determine whether the specific phenotypes of hyperandrogenism and ovulatory dysfunction associated with PCOS influence the efficacy of such combined interventions. Consequently, bridging this evidence gap is crucial for clarifying the biological mechanisms of combined interventions and advancing precision nutritional therapy for PCOS (Table 2).

**Table 2:** Summary of clinical studies on probiotic-related interventions in women with PCOS.

Study (Ref.)	Design and participants	Intervention type	Intervention, dose, and co-intervention	Control	Duration	Primary/major endpoints	Main findings
Zhang et al., 2019 (81)	Two-phase study; probiotic intervention phase included 14 women with PCOS. BMI/PCOS phenotype not reported for the intervention subgroup; no parallel randomized control.	Single-strain probiotic	*Bifidobacterium animalis* subsp*. lactis* V9 (reported as *B. lactis* V9), (4 × 10^10^ CFU/day), once daily.	None in the 10-week probiotic intervention phase.	10 weeks	Exploratory monitoring of gut microbiota, strain colonization, SCFAs, gut-brain mediators, LH, LH/FSH ratio, and sex hormones.	Among 9 participants with effective colonization, LH and the LH/FSH ratio decreased, while SCFA concentrations and several sex-hormone-related measures increased. Findings were from a small uncontrolled intervention subgroup.
Karamali et al., 2018 (82)	Randomized, double-blind, placebo-controlled trial; 60 women with PCOS, aged 18–40 years (30/group). BMI/phenotype not clearly specified in the report.	Multi-strain probiotic	*Lactobacillus acidophilus*, *Lacticaseibacillus casei*, and *Bifidobacterium bifidum*; reported concentration 2 × 10^9^ CFU/g for each strain.	Matching placebo.	12 weeks	Hormonal profiles and clinical hyperandrogenism; inflammatory and oxidative-stress biomarkers.	Compared with placebo, total testosterone, modified Ferriman–Gallwey score, hs-CRP, and MDA decreased; SHBG and TAC increased. HOMA-IR and other metabolic measures showed no significant between-group improvement.
Darvishi et al., 2021 (83)	Randomized, double-blind, placebo-controlled trial; 68 overweight or obese women with PCOS, aged 20–44 years (34/group).	Synbiotic	One 500-mg capsule/day containing *Lacticaseibacillus casei*, *Lacticaseibacillus rhamnosus*, *Lactobacillus delbrueckii* subsp*. bulgaricus*, *Lactobacillus acidophilus*, *Bifidobacterium longum*, *Bifidobacterium breve*, *Streptococcus thermophilus*, plus inulin-type fructooligosaccharides.	Starch placebo capsule.	8 weeks	Glycemic indices, lipid profile, serum apelin, body weight, BMI, and central-adiposity measures.	Fasting glucose, insulin, HOMA-IR, body weight, BMI, waist and hip circumferences, and waist-to-height ratio decreased; HDL cholesterol increased. Total cholesterol, triglycerides, LDL cholesterol, apelin, and waist-to-hip ratio showed no significant between-group change.
Ghanei et al., 2018 (89)	Randomized, double-blind, placebo-controlled trial; 90 women screened, 70 entered the protocol, and 60 completed; Rotterdam-diagnosed PCOS, aged 18–40 years. BMI not clearly reported.	Multi-strain probiotic with drug co-intervention	*Lactobacillus acidophilus*, *Lactiplantibacillus plantarum*, *Limosilactobacillus fermentum*, and *Lactobacillus gasseri*; reported concentration 1 × 10^9^ CFU/g. Both groups also received cyproterone acetate.	Maltodextrin placebo plus the same cyproterone-acetate regimen.	12 weeks	Inflammatory markers (IL-6, IL-10, TNF-α, hs-CRP) and anthropometric/clinical measures.	IL-10 increased significantly with probiotics. hs-CRP and IL-6 decreased in both groups, whereas TNF-α showed no significant probiotic-specific change. Interpretation should account for concurrent cyproterone-acetate therapy.
Ji et al., 2022 (90)	Single-center randomized three-arm trial; 60 non-obese women with PCOS, allocated 1:1:1.	Probiotic alone, metformin alone, and combined probiotic + metformin	ProMetS probiotic powder, 4 g/day; metformin, 1.5 g/day; or their combination. Strain composition was not reported in the accessible article abstract/registry.	Active-comparator design: probiotic alone, metformin alone, and combination; no placebo group.	12 weeks	Primary: improvement in menstrual patterns. Secondary: ovulation, anthropometric measures, metabolic profiles, and hormonal levels.	Menstrual-cycle recovery was 40, 55, and 80%, and ovulation was 30, 55, and 75%, in probiotic, metformin, and combination groups, respectively. The combination was superior for menstrual outcomes; BMI, fasting glucose, HOMA-IR, lipids, AMH, testosterone, and FAI also improved after probiotic and/or metformin treatment.
Jamilian et al., 2018 (91)	Randomized, double-blind, placebo-controlled trial; 60 women with Rotterdam-diagnosed PCOS, aged 18–40 years (30/group). BMI/phenotype not clearly specified.	Multi-strain probiotic + selenium co-supplementation	*Lactobacillus acidophilus*, *Limosilactobacillus reuteri*, *Limosilactobacillus fermentum*, and *Bifidobacterium bifidum* (2 × 10^9^ CFU/g each; total 8 × 10^9^ CFU/day) plus selenium 200 μg/day.	Matching placebo.	12 weeks	Primary: hormonal profiles. Secondary: mental-health measures, inflammation, and oxidative-stress biomarkers.	Compared with placebo, BDI, GHQ, and DASS scores improved; total testosterone, hirsutism score, hs-CRP, and MDA decreased; TAC and GSH increased. Because probiotics and selenium were co-administered, their individual contributions cannot be separated.
Szydłowska et al., 2025 (92)	Randomized, double-blind, placebo-controlled trial; 50 women with Rotterdam-diagnosed PCOS (25/group), 43 completed. Mean age 28.4 years; mixed BMI, with potential relevance to overweight/high-FAI phenotypes.	Multi-strain probiotic	SanProbi Barrier: *Bifidobacterium bifidum* W23, *Bifidobacterium animalis* subsp*. lactis* W52 and W51, *Lactobacillus acidophilus* W37, *Levilactobacillus brevis* W63, *Lacticaseibacillus casei* W56, *Ligilactobacillus salivarius* W24, and *Lactococcus lactis* W19/W58; 1 × 10^9^ CFU/day.	Matching placebo.	12 weeks	Hormone concentrations and BMI.	Within the probiotic group, LH, TSH, androstenedione, and BMI decreased, while SHBG increased. Between-group delta changes were significant for TSH, androstenedione, SHBG, and BMI; LH reached significance only with a one-tailed test. Testosterone and DHEAS did not show significant between-group improvement.
Chudzicka-Strugała et al., 2025 (93)	Randomized, placebo-controlled trial; 70 overweight/obese women invited, 65 randomized, and 33 completed 6 months; BMI > 25 kg/m^2^; Rotterdam PCOS.	Synbiotic + intensive lifestyle modification	SANPROBI Super Formula, four capsules/day: *Bifidobacterium animalis* subsp*. lactis* W51/W52, *Lactobacillus acidophilus* W22, *Lacticaseibacillus paracasei* W20, *Lactiplantibacillus plantarum* W21, *Ligilactobacillus salivarius* W24, *Lactococcus lactis* W19, plus fructooligosaccharides and inulin. Both groups followed a 1,400–1800 kcal/day diet and daily walking.	Four placebo capsules/day plus the same intensive lifestyle program.	6 months	Primary: BMI, body composition, and total testosterone. Additional endocrine, metabolic, lipid, and endotoxemia markers.	BMI and body fat decreased similarly in both groups. Synbiotic supplementation produced greater reductions in total testosterone, LH, total cholesterol, LDL cholesterol, triglycerides, LPS, and LBP; fasting insulin decreased and ISI increased in the synbiotic group.
Shirani et al., 2025 (94)	Parallel, double-blind, placebo-controlled randomized trial; 90 women with PCOS, 86 completed. Participants were stratified by BMI < 25 or ≥25 kg/m^2^.	Two-strain probiotic	*Lactobacillus helveticus* R0052 and *Bifidobacterium longum* R0175, total 3 × 10^9^ CFU/day, one capsule before lunch; usual medical care was allowed.	Matching 300-mg maltodextrin placebo plus usual care.	8 weeks	Hormonal status, oxidative stress, CRP, and clinical symptoms; SHBG was the key variable used for sample-size calculation.	SHBG, TAC, and SOD increased; FAI, CRP, and MDA decreased. Total testosterone was not significantly different between groups after adjustment. Acne, alopecia, and hirsutism did not show significant adjusted between-group improvement.

HOMA-IR, homeostatic model assessment of insulin resistance; CRP, C-reactive protein; LH, luteinizing hormone; FSH, follicle-stimulating hormone; SHBG, sex hormone-binding globulin; hs-CRP, high-sensitivity C-reactive protein; IL-6, interleukin-6; TNF-α, tumor necrosis factor-α; MDA, malondialdehyde; TAC, total antioxidant capacity; GSH, glutathione; ISI, insulin sensitivity index; LPS, lipopolysaccharide; LBP, lipopolysaccharide-binding protein; DHEAS, dehydroepiandrosterone sulfate; FAI, free androgen index.

## 5 Discussion

### 5.1 Comprehensive interpretation of key findings

This review systematically integrates hierarchical evidence ranging from fundamental mechanisms to clinical translation, centering on the theme of the “KD–gut microbiota–PCOS” axis. The key findings can be summarized in the following four aspects:

First, the gut–immune microenvironment represents a convergent pathogenic node in PCOS and immune-related gastrointestinal disorders. PCOS-related gut dysbiosis recapitulates key immunopathological features of IBD—including barrier dysfunction, Treg/Th17 imbalance, and metabolite-driven mucosal inflammation—positioning nutritional modulation of the gut immune microenvironment as a transdiagnostic therapeutic strategy.

Second, the KD is an effective dietary intervention strategy for PCOS, yet its effects are mediated by the gut microbiota and are subject to “microbiota-related limitations.” Multiple RCTs and three recent meta-analyses consistently confirm the efficacy of KD in improving metabolic and reproductive parameters in PCOS. However, a landmark study by Ang et al. demonstrated that β-HB directly inhibits the growth of *Bifidobacterium*, revealing the “dual nature” of KD’s impact on the gut microbiota. Meanwhile, the discovery of the GDF15-GFRAL pathway by Lu et al. provides a novel framework for understanding the mechanisms underlying KD-induced weight loss.

Third, probiotics serve as an important adjunctive therapy for PCOS. A meta-analysis by Li et al. covering 17 RCTs and 1,049 participants, confirmed that probiotics, prebiotics, and synbiotics can significantly improve insulin resistance (IR) and lipid profiles in patients with PCOS. He et al. discovered that *Lp. plantarum* CCFM1019 alleviates PCOS via a butyrate-dependent gut–brain axis mechanism, offering new insights into the molecular mechanisms of probiotics.

Fourth, KD and probiotics may act through potentially complementary mechanisms, but current support is based mainly on indirect evidence. No randomized controlled trial has directly evaluated their combined use in women with PCOS; therefore, any additional benefit requires clinical validation. The proposed MR–MR–IS framework and five-level model should be regarded as conceptual frameworks for future investigation.

From a clinical perspective, gastrointestinal outcomes should be interpreted separately from core PCOS outcomes. Improvements in constipation, bloating, abdominal discomfort, or other gastrointestinal symptoms primarily reflect local gastrointestinal effects and may also provide information regarding treatment tolerability and adherence. In contrast, clinically meaningful improvement in PCOS should be evaluated using disease-specific outcomes, including insulin resistance, androgen-related indicators, menstrual regularity, and ovulation. Gastrointestinal symptoms therefore remain relevant secondary outcomes, but improvements in these symptoms alone should not be interpreted as evidence of improvement in the underlying metabolic and reproductive-endocrine features of PCOS. Endocrine and reproductive outcomes should also be interpreted according to distinct physiological domains. Androgen-related outcomes include total or free testosterone, DHEAS, SHBG, FAI, and clinical hyperandrogenism; gonadotropin-related outcomes include LH, FSH, and the LH/FSH ratio; and reproductive outcomes include menstrual regularity, ovulation, and pregnancy. Improvements in one domain should not be generalized as evidence of improvement across all endocrine and reproductive outcomes, particularly because several findings are derived from small studies.

### 5.2 Analysis of the sources of evidence heterogeneity

The heterogeneity observed in the existing evidence stems primarily from the following aspects:

1. Differences in PCOS diagnostic criteria: Studies have employed the Rotterdam, NIH, or Androgen Excess and PCOS Society (AE-PCOS) criteria, resulting in significant variations in the clinical phenotypes of the enrolled populations.
2. Diversity of KD formulations: Studies differ regarding the degree of carbohydrate restriction (<20 g vs. <50 g/day), protein proportion (15–25%), and fat composition (predominantly saturated fat vs. increased omega-3 intake); these differences in formulation may affect both gut microbial composition and clinical outcomes.
3. High heterogeneity of probiotic strains: Different strains vary widely in their efficacy regarding the improvement of metabolic, hormonal, and inflammatory parameters. A subgroup analysis by Li suggested that multi-strain probiotic formulations may be more effective than single-strain products, although the most appropriate strain combination remains unclear.
4. Inconsistent findings on SCFA levels: da Silva et al. reported increases in acetate and propionate, whereas most other studies found lower SCFA levels. This inconsistency may be related to differences in dietary background, BMI, and measurement methods. In individuals with excess energy intake and insulin resistance, altered fermentation by certain gut bacteria, such as *Prevotella copri*, may increase acetate and propionate levels. In this setting, higher SCFA levels may reflect microbial imbalance rather than a beneficial metabolic response.

### 5.3 Limitations and challenges of current evidence

1. Lack of direct evidence from randomized controlled trials: No randomized controlled trial has yet examined the combined use of a ketogenic diet and probiotics in women with PCOS. Although studies in obesity and epilepsy provide some indirect support, these findings cannot be directly applied to PCOS. The effects of this combined approach on hyperandrogenism, ovulatory dysfunction, and other PCOS-related features still need to be tested in well-designed clinical trials.
2. Limited data on changes in the gut microbiota: Published clinical studies of ketogenic diets in women with PCOS have generally not included gut microbiota analysis. As a result, it remains unclear how the diet affects microbial composition and whether these changes are related to improvements in metabolic, hormonal, or reproductive outcomes.
3. Insufficient long-term safety data: Most studies involved interventions lasting 12 weeks or less. There is limited evidence regarding the long-term cardiovascular safety of KD, its effects on bone density and thyroid function, and the safety of prolonged probiotic use in women with PCOS. Potential concerns associated with ketogenic diets also include inadequate dietary fiber intake, constipation, gastrointestinal discomfort, micronutrient insufficiency, and unfavorable lipid responses in susceptible individuals. Future clinical trials should systematically report gastrointestinal adverse events, lipid profiles, dietary adherence, withdrawal rates, and long-term safety outcomes.
4. Difficulties in developing personalized interventions: Although PCOS treatment may need to be tailored to different clinical phenotypes, it is still difficult to apply this approach in routine practice. More evidence is needed to clarify how specific bacterial strains and metabolic patterns are related to different PCOS features before practical treatment protocols can be developed.

### 5.4 Future research directions

1. Conduct high-quality trials of combined ketogenic diet and probiotic interventions: Future studies should directly compare a ketogenic diet alone, a ketogenic diet combined with probiotics, and a control intervention in women with PCOS. A three-arm randomized controlled trial with at least 180 participants and a 12-week intervention period could assess changes in metabolic, microbial, inflammatory, and reproductive outcomes.
2. Use a standardized core outcome set: Future dietary intervention studies in PCOS should use consistent primary and secondary outcomes. This would make it easier to compare findings across trials and evaluate the overall effectiveness of different interventions.
3. Integrate multi-omics approaches: Combining metagenomic, metabolomic, and host transcriptomic data may provide a more complete understanding of the interactions among diet, the gut microbiota, microbial metabolites, and host responses.
4. Validate biomarker-guided personalized therapy: Prospectively investigate whether baseline microbiota characteristics (e.g., alpha diversity, *Bacteroides*/*Prevotella* ratio, and SCFA levels) can predict the response to KD or probiotic treatment.
5. Explore next-generation probiotics and postbiotics: Future studies may also examine newer probiotic candidates, such as *Akkermansia muciniphila* and *Faecalibacterium prausnitzii*, as well as postbiotic products including slow-release butyrate, inactivated probiotics, and microbial metabolites. These approaches may provide more consistent and targeted options for managing PCOS.
6. Compare findings with IBD populations: Further studies could compare the effects of combined ketogenic diet and probiotic interventions in patients with PCOS and IBD. This may help determine whether the two conditions share similar changes in the gut microbiota and immune environment, and whether common biomarkers can be used to guide more individualized nutritional interventions.

## 6 Conclusion and future perspectives

In summary, the roles of the KD and probiotic intervention in PCOS management focus on two key aspects—metabolic regulation and the gut microbiota—and may have complementary mechanisms of action. The KD may improve insulin resistance, support weight loss, and regulate glucose and lipid metabolism by restricting carbohydrate intake. Probiotics, in contrast, may improve metabolic and endocrine function by modifying the gut microbiota, influencing microbial metabolites, strengthening the intestinal barrier, and reducing chronic inflammation. Combining these two approaches may therefore provide complementary benefits by addressing both metabolic dysfunction and gut microbial imbalance in PCOS.

However, no randomized controlled trial has directly evaluated the combined use of a ketogenic diet and probiotics in women with PCOS. Therefore, the proposed complementary effects remain hypothetical and require clinical validation. First, large, well-designed randomized controlled trials examining the combined use of a ketogenic diet and probiotics in women with PCOS are still lacking. In addition, there is significant heterogeneity regarding PCOS patient subtypes, intervention protocols, and microbiota analysis strategies. The absence of standardized methods for microbiota analysis (e.g., 16S rRNA sequencing vs. metagenomics), KD types, and probiotic strain selection limits the comparability of results. Finally, systematic data regarding the safety and adherence associated with long-term interventions, as well as inter-individual differences in response to combined therapy, remain insufficient, thereby hindering widespread clinical adoption.

Future research should focus on the following directions. First, prospective multicenter randomized controlled trials are needed to compare combined ketogenic diet and probiotic therapy with either intervention alone. These studies should assess metabolic, endocrine, and reproductive outcomes in women with PCOS and determine whether the combined approach offers any additional benefit. Second, further research is needed to determine how the combined intervention changes the gut microbiota over time and whether these changes differ among women with different PCOS phenotypes, including obesity, insulin resistance, and hyperandrogenism. Identifying microbial features linked to treatment response may also help predict which patients are more likely to benefit. Third, by integrating multiomics technologies (such as metagenomics, metabolomics, and transcriptomics), the molecular network mechanisms underlying the combined regulation of gut microbiota and their metabolites by KDs and probiotics can be elucidated, thereby providing a scientific basis for precision interventions. Fourth, attention must be paid to the safety of long-term interventions—specifically, whether the sustained inhibitory effect of β-HB on *Bifidobacterium* necessitates periodic probiotic supplementation to maintain microbial balance—to provide comprehensive evidence supporting clinical implementation.

Future research may help refine precision nutrition approaches for PCOS by examining GDF15–GFRAL signaling, the interaction between β-HB and the gut microbiota, and the broader gut–brain–ovary axis. Combining metabolic evaluation with microbial profiling may allow dietary interventions to be better matched to the clinical characteristics of individual patients, which could improve treatment response and support better long-term outcomes.
